# ﻿Review of species of the genus *Heterospilus* Haliday, 1836 (Hymenoptera, Braconidae, Doryctinae) from the Korean Peninsula

**DOI:** 10.3897/zookeys.1079.73701

**Published:** 2021-12-22

**Authors:** Sergey A. Belokobylskij, Deok-Seo Ku

**Affiliations:** 1 Zoological Institute, Russian Academy of Sciences, St Petersburg 199034, Russia Zoological Institute, Russian Academy of Sciences St Petersburg Russia; 2 Museum and Institute of Zoology, Polish Academy of Sciences, Wilcza 64, Warszawa 00–679, Poland Museum and Institute of Zoology, Polish Academy of Sciences Warszawa Poland; 3 The Science Museum of Natural Enemies, Geochang 50147, South Korea The Science Museum of Natural Enemies Geochang Republic of Korea

**Keywords:** Descriptions, Ichneumonoidea, key, new records, new species, parasitoid

## Abstract

This article reviews the species of the genus *Heterospilus* Haliday from South Korea. Nine species, *Heterospiluschinjuensis***sp. nov.**, *H.gajwaensis***sp. nov.**, *H.heulriensis***sp. nov.**, *H.hyungkeunleei***sp. nov.**, *H.maseongus***sp. nov.**, *H.suriensis***sp. nov.**, *H taehoan*i **sp. nov.**, *H.weolchulsanus***sp. nov.**, and *H.yeogiensis***sp. nov.**, are described as new to science. The species *Heterospilusfujianensis* Tang, Belokobylskij, He & Chen, 2013 is recorded for the fauna of Korea for the first time; *H.ater* Fischer, 1960 is synonymised under *H.austriacus* (Szépligeti, 1906). *Heterospilusrubicola* Fischer, 1960 and *H.corsicus* (Marshall, 1888) are excluded from the fauna of Korea. A key to all *Heterospilus* species known from the Korean Peninsula is compiled.

## ﻿Introduction

The Doryctinae genus *Heterospilus* Haliday, 1836 from the tribe Heterospilini, together with *Spathius* Nees, 1819 (Spathiini), is one of the largest and hyperdiverse genera in the subfamily Doryctinae, including numerous undescribed yet species (Belokobylskij and Maetô 2009; Yu et al. 2016). According to the last molecular phylogenetic analysis provided for the subfamily Doryctinae (Zaldivar-Riveron et al. 2008), this genus possibly originated in the tropics of South America with its later penetration and wide distribution in Old World continents.

In total, 413 valid species of this genus have been described worldwide (Marsh et al. 2013; Tang et al. 2013; Yu et al. 2016), and more than half (280) were recorded only in the New World (Costa Rica) (Marsh et al. 2013). The actual number of species in this genus is probably much larger because of not enough investigated the tropical (predominantly South American) *Heterospilus* fauna. Two subgenera, *Eoheterospilus* Belokobylskij & Maetô, 2009 (with only two known species) and the nominative *Heterospilus* s. str. (with all the remaining species), are recognised in this genus (Belokobylskij and Maetô 2009).

The study of *Heterospilus* fauna in Eastern Asian countries started relatively recently (Belokobylskij 1983, 1994, 1996; 1998; Belokobylskij and Maetô 2009; Tang et al. 2013). Thus far, fourteen *Heterospilus* species have been recorded in the Russian Far East, 34 species in China (including Taiwan), 24 species in Japan (including Ryukyu and Ogasawara Islands), and only five species are known in Vietnam.

The genus *Heterospilus* in the Korean Peninsula is not abundant and previously 15 species have been recorded in this territory: *H.ater* Fischer, 1960; *H.austriacus* (Szépligeti, 1906); *H.cephi* Rohwer, 1925; *H.chinensis* Chen & Shi, 2004, *H.corsicus* (Marshall, 1888); *H.extasus* Papp, 1987; *H.kerzhneri* Belokobylskij & Maeto, 2009; *H.leptosoma* Fischer, 1960; *H.orientalis* Belokobylskij, 1983; *H.rubicola* Fischer, 1968; *H.rubrocinctus* (Ashmead, 1905); *H.separatus* Fischer, 1960; *H.tauricus* Telenga, 1941; *H.tirnax* Papp, 1987; *H.zaykovi* van Achterberg, 1992 (Papp 1987, 1992; Ku et al. 2001; Belokobylskij and Maetô 2009; Lee et al 2020). However, the records the species *H.rubicola* Fischer (see Remarks under *H.kerzhneri* Belokobylskij & Maeto) and *H.corsicus* (Marshall) are questionable and require reconfirmation. At least re-study of the available specimens determined as *H.corsicus* showed that it does not belong to this species and they belong to several other species (*H.chinensis* Chen & Shi, 2004, *H.separatus* Fischer, 1960, etc.). The status of *H.corsicus* and its distribution in the Eastern Palaearctic require a separate additional study and the former records need reconfirmation. As a result, *H.corsicus* (Marshall, 1888) is excluded here from the fauna of the Korean Peninsula.

In this paper, nine species of *Heterospilus* Haliday are described from Korea as new to science, and a single additional species is recorded for the first time. A key to all known Korean species is provided.

## ﻿Materials and methods

The terminology employed for the morphological features, sculpture and body measurements follows Belokobylskij and Maetô (2009). The wing venation nomenclature follows Belokobylskij and Maetô (2009), with the terminology of van Achterberg (1993) shown in parentheses. New distribution records presented in this paper are marked with an asterisk (*).

The specimens were examined using an Olympus SZ51 stereomicroscope. Photographs were taken with an Olympus OM-D E-M1 digital camera mounted on an Olympus SZX10 microscope (Zoological Institute of the Russian Academy of Sciences, St. Petersburg, Russia). Image stacking was performed using Helicon Focus 5.0. The figures were created using the Adobe Photoshop CS6 program.

The specimens examined in this study were deposited in the collections of the Hungarian Natural History Museum (Budapest, Hungary; **HNHM**), the Naturhistorische Museum Wien (Wien, Austria; **NHMW**), the Natural History Museum (London, United Kingdom; **NHMUK**), the National Institute of Biological Resources (Incheon, Republic of Korea; **NIBR**), the Science Museum of Natural Enemies (Geochang, Republic of Korea; **SMNE**), and the Zoological Institute of the Russian Academy of Sciences (St Petersburg, Russia; **ZISP**).

## ﻿Taxonomy

**Class Hexapoda Blainville, 1816**

**Order Hymenoptera Linnaeus, 1758**

**Family Braconidae Nees, 1811**

**Subfamily Doryctinae Foerster, 1863**

**Tribe Heterospilini Fischer, 1981**

**Genus *Heterospilus* Haliday, 1836**

### ﻿Updated checklist of *Heterospilus* species recorded in the fauna of the Korean peninsula

Heterospilus (Eoheterospilus) rubrocinctus (Ashmead, 1905)

Heterospilus (Heterospilus) austriacus (Szépligeti, 1906) (= *H.ater* Fischer, 1960, syn. nov.)

Heterospilus (Heterospilus) cephi Rohwer, 1925

Heterospilus (Heterospilus) chinensis Chen & Shi, 2004

Heterospilus (Heterospilus) chinjuensis sp. nov.

Heterospilus (Heterospilus) extasus Papp, 1987

Heterospilus (Heterospilus) fujianensis Tang, Belokobylskij, He & Chen, 2013

Heterospilus (Heterospilus) gajwaensis sp. nov.

Heterospilus (Heterospilus) heulriensis sp. nov.

Heterospilus (Heterospilus) hyungkeunleei sp. nov.

Heterospilus (Heterospilus) kerzhneri Belokobylskij & Maetô, 2009

Heterospilus (Heterospilus) leptosoma Fischer, 1960

Heterospilus (Heterospilus) maseongus sp. nov.

Heterospilus (Heterospilus) orientalis Belokobylskij, 1983

Heterospilus (Heterospilus) separatus Fischer, 1960

Heterospilus (Heterospilus) suriensis sp. nov.

Heterospilus (Heterospilus) taehoani sp. nov.

Heterospilus (Heterospilus) tauricus Telenga, 1941

Heterospilus (Heterospilus) tirnax Papp, 1987

Heterospilus (Heterospilus) weolchulsanus sp. nov.

Heterospilus (Heterospilus) yeogiensis sp. nov.

Heterospilus (Heterospilus) zaykovi van Achterberg, 1992

#### ﻿ Heterospilus (Heterospilus) austriacus

Taxon classificationAnimaliaHymenopteraBraconidae

(Szépligeti, 1906)

6DFB6FE5-90B7-5BC3-8E5D-D97D33C13B00

Figs 1 , 2

Atoreuteus austriacus Szépligeti, 1906: 605; Papp, 1984: 177 (as synonym of H.sicanus (Marshall)).
Heterospilus austriacus : Belokobylskij and Tobias 1986: 34; Yu et al. 2016; Tang et al. 2013: 2002 (as valid species).
Heterospilus ater Fischer, 1960: 36. Syn. nov.

##### Type material examined.

Lectotype of *Atoreuteusaustriacus* Szépligeti, 1906: female, “Austria, Wien Umgbg”, “Wien Umgbg”, “Lectotypus ♀ *Atoreuteusaustriacus* Szépl. 1906., des. Papp J., 1969”, “Hym. Typ. No 1650 Mus. Budapest”, “♀ *Heterospilusaustriacus* (Szépl.), Det. C. v. Achterberg, 1979” (HNHM). Holotype of *Heterospilusater* Fischer, 1960: female, [Austria] “St. Marx, Wien, 24.5.[19]59, leg. Fischer”, “*Heterospilusater* n. sp., det. Fischer”, “Holotype” (NHMW).

##### Additional material examined.

South Korea: 1 female, Kyŏnggi, Suwon, Mt. Yŏgi, 11.V.1994, D.-S. Ku leg. (NIBR); 1 female, Seoul-si, Hongrung, Forestry Research Institute, light trap, VII.1998, S.-H. Gang leg.; 1 female, Jeonnam-do, Yeongam-gun, Gunseo-myeon, Dogap-ri, Temple Dogapsa (Mt. Weolchulsan), light trap, 24–25.VII.1990, J.-S. Jeon leg.; 1 female, Gyeongsangnam-do, Sancheong-gun, 30 km NNW of Jinju (Chinju), forest, h = 800 m, 12.VI.2002, S. Belokobylskij leg.

##### Distribution.

Korean Peninsula (Ku et al. 2001); China, Russia (European part, Urals, Siberia, Far East), Kazakhstan, Transcaucasia, Central and Western Europe (Yu et al. 2016; Belokobylskij et al. 2019).

##### Remarks.

Species *Atoreuteusaustriacus* Szépligeti, 1906 was synonymised under *Dentrosotersicanus* Marshall, 1888 by J. Papp (1984, 2004). However, our study of the types specimens of both species showed that *H.austriacus* differs distinctly from *H.sicanus* (holotype: female, with labels “Type” (round with red ringing), “Marshall coll. 1904-120”, *sicanus* Marsh. (Sicily)”, “Almost certainly type of *Dendrosotersicanus* Msh., G. Nixon, 25.I.38” (handwriting by Nixon), “B.M. Type Hym. 3c.1751” (NHMUK)) by the short ovipositor sheath, which is 0.3–0.4× as long as the metasoma, 0.5–0.6× as long as the mesosoma and 0.2–0.3× as long as the fore wing (long, 0.8× as long as the metasoma, 1.1× longer than the mesosoma and 0.5× as long as the fore wing in *H.sicanus*), long and entirely sculptured second metasomal tergite (second tergite short and smooth laterally), mesopleuron smooth medially on wide area (almost entirely rugose-striate with granulation in *H.sicanus*), and vertex weakly and interruptedly transverse striate (entirely and densely striate in *H.sicanus*). As a result, this species name was recently restored as a valid species *Heterospilusaustriacus* (Szépligeti) from the synonymy of *H.sicanus* (Tang et al. 2013).

**Figure 1.**
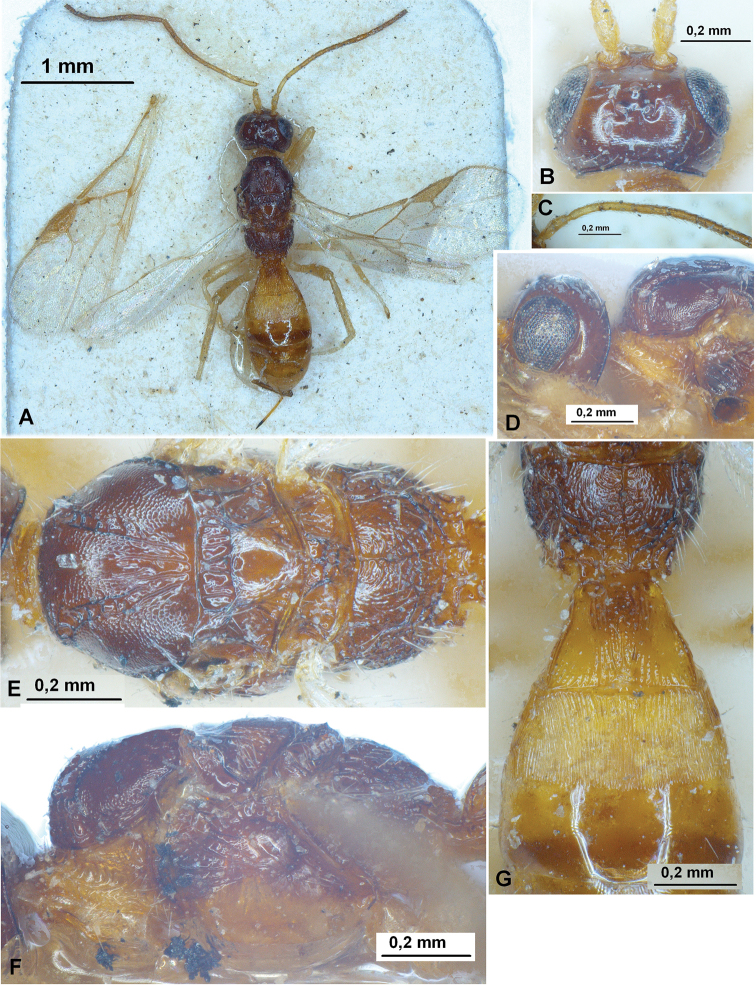
Heterospilus (Heterospilus) austriacus (Szépligeti, 1906), female, lectotype **A** habitus, dorsal view **B** head, dorsal view **C** basal segments of antenna **D** head and anterior part of mesosoma, lateral view **E** mesosoma, dorsal view **F** mesosoma, lateral view **G** propodeum and three basal tergites of metasoma, dorsal view

The study of the large *Heterospilus* material and type of specimens belonging to *H.austriacus* and *H.ater* distinctly showed significant variability in the range of the intensity of the subbasal transverse furrow and its sculpture on the third metasomal tergite, the main diagnostic character of the discussed species. As a result, *Heterospilusater* Fischer, 1960 is here synonymised under *H.austriacus* (Szépligeti, 1906), syn. nov.

**Figure 2.**
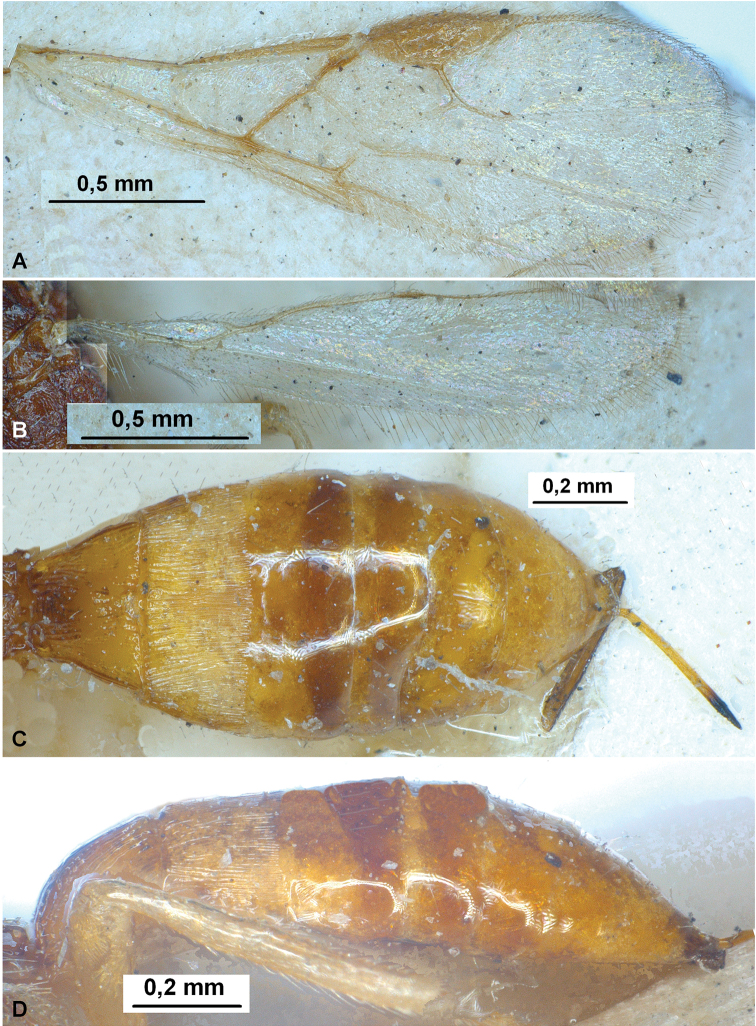
Heterospilus (Heterospilus) austriacus (Szépligeti, 1906), female, lectotype **A** fore wing **B** hind wing **C** metasoma, dorsal view **D** metasoma, lateral view

#### ﻿ Heterospilus (Heterospilus) chinensis

Taxon classificationAnimaliaHymenopteraBraconidae

Chen & Shi, 2004

A43AB1FE-4541-53BC-845E-5B3FA3FCA25C

Heterospilus chinensis Chen & Shi, 2004: 72 Yu et al. 2016; Lee et al. 2020: 19.

##### Material examined.

South Korea: 1 female, Suwon, Mt. Yeoki, MT (Wh/Gr), 21.VII.1997, J.-Y. Choi leg.; 3 females, Seoul-si, Hongrung, Forestry Research Institute, light trap, VII.1998, S.-H. Gang leg.; 1 female, Gyeongbuk-do, Pohang-si, Songla-myeon, Daejeon-ri, Saryeongjeon (Mt. Naeyeonsan), sweeping, 16.VIII.1997, J.-S. Jeon leg.; 1 female, Gyeongnam-do, Jinju-si, Gajwa-dong, Malaise trap, 30.VI–7.VII.1987, D.-S. Ku leg.

##### Distribution.

Korean Peninsula; China, Japan.

#### ﻿ Heterospilus (Heterospilus) chinjuensis sp. nov.

Taxon classificationAnimaliaHymenopteraBraconidae

8EE13CAC-5632-5FC8-BBA6-034568EF8250

http://zoobank.org/3C088A6F-D140-4C61-B2D1-41BD4AEA58F1

Figs 3 , 4

##### Type material.

***Holotype***. female, “Korea, Kyongnam, Chinju-shi, Kajwadong, 1.VI.1993, Deok-Seo Ku” (NIBR).

***Paratype.*** 1 female, “Korea, KyongNam, Chinju, Chojeon-dong (at Mercury lamp), 8–9. VIII. 1995, Deok-Seo Ku” (SMNE).

##### Comparative diagnosis.

This species is very similar to *H.taehoani* sp. nov., but differs from the latter by having the POL 0.7× Od (1.0–1.3× in *H.taehoani*), hind femur wider, 3.6× longer than wide (slender, 4.0–4.4× longer than wide in *H.taehoani*), length of the first tergite 1.15× its distal width (almost equal in *H.taehoani*) and second tergite entirely striate (striate only in the basal half in *H.taehoani*).

**Figure 3.**
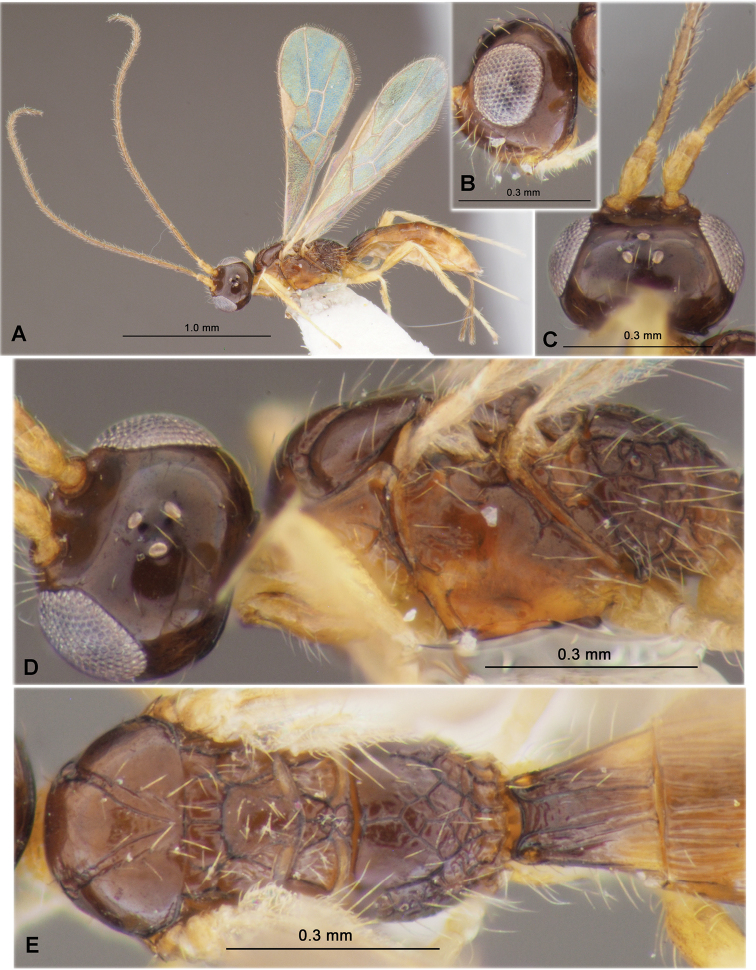
Heterospilus (Heterospilus) chinjuensis sp. nov., female, holotype **A** habitus, lateral view **B** head, lateral view **C** head, dorsal view **D** head in dorsolateral view and mesosoma in lateral view **E** mesosoma and first metasomal tergite, dorsal view

##### Description.

**Female.** Body length 2.0 mm; fore wing length 1.6–1.7 mm.

***Head.*** Head not depressed, its width (dorsal view) 1.4–1.6× median length, 1.2–1.3× width of mesoscutum. Head behind eyes (dorsal view) distinctly and regularly curvedly narrowed; transverse diameter of eye 1.6–1.7× longer than temple. Ocelli small, arranged in triangle with base 1.2× its sides. POL 0.7–0.8× Od, 0.3–0.4× OOL. Diameter of antennal socket 1.1–1.3× distance between sockets, 1.4–1.7× distance between socket and eye. Eye without setae, with very shallow emargination opposite antennal sockets, 1.15× as high as broad. Malar space 0.4× height of eye, 1.0–1.2× basal width of mandible. Face weakly convex, its width almost equal to eye height and 1.2–1.3× height of face and clypeus combined. Hypoclypeal depression medium-sized and oval, its width 0.8–0.9× distance from edge of depression to eye, 0.4–0.5× width of face. Occipital carina joined ventrally with hypostomal carina distinctly above base of mandible. Head below eyes (front view) distinctly and roundly narrowed.

**Figure 4.**
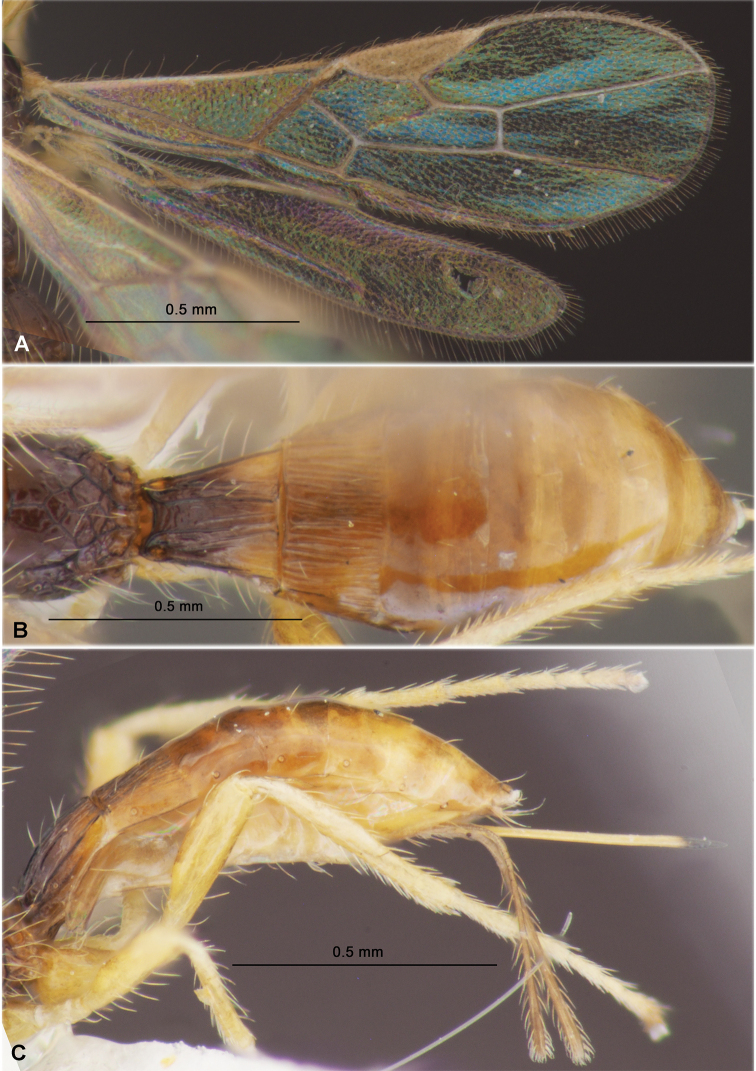
Heterospilus (Heterospilus) chinjuensis sp. nov., female, holotype **A** wings **B** propodeum and metasoma, dorsal view **C** metasoma and hind leg, lateral view

***Antenna***. Antenna slender, filiform, 20-segmented, ~ 1.2× longer than body. Scape short and rather thick, 1.2–1.4× longer than its maximum width. First flagellar segment slender, almost straight, subcylindrical, 4.5–5.5× longer than its apical width, 0.9–1.0× as long as second segment. Penultimate segment 4.5× longer than wide, 0.8× as long as first flagellar segment and 1.0–1.1× as long as apical segment; the latter acuminate apically and without spine.

***Mesosoma***. Mesosoma not depressed, its length 1.8–2.0× maximum height. Pronotum elongated, dorsally weakly convex (lateral view). Mesoscutum distinctly and almost perpendicularly elevated above pronotum (lateral view), maximum width of mesoscutum (dorsal view) 1.1× its length. Median lobe of mesoscutum weakly protruding forwards, without anterolateral corners, convex anteriorly (dorsal view). Notauli narrow, entirely deep, mainly smooth, with sparse and fine crenulae. Prescutellar depression deep and long, with three high median carinae, smooth on remaining places, 0.6× as long as scutellum. Scutellum weakly convex, without lateral carinae, its basal width almost equal to median length. Subalar depression rather shallow, wide, with few and sparse curved striae medially, mainly smooth. Precoxal sulcus distinct, almost straight, completely smooth, running along anterior half of lower part of mesopleuron. Metanotal dorsal tooth very low, wide, subpointed (lateral view). Metapleural lobe short, wide, rounded apically. Propodeum without lateral tubercles.

***Wings***. Fore wing 3.0–3.5× longer than its maximum width. Pterostigma 4.0–4.5× longer than wide. Metacarp (1-R1) 1.4× longer than pterostigma. Radial vein (r) arising from middle of pterostigma. First radial abscissa (r) 0.9–1.0× as long as maximum width of pterostigma. Second radial abscissa (3-SR) 1.7× longer than first abscissa (r) and forming with it obtuse angle, 0.3× as long as almost straight third abscissa (SR1), 0.6–0.8× as long as trace of first radiomedial vein (2-SR). Trace of first radiomedial vein (2-SR) 2.3–2.5× longer than second radiomedial vein (r-m) and 2.7–3.2× longer than recurrent vein (m-cu). Recurrent vein (m-cu) postfurcal. First medial abscissa (1-SR+M) weakly curved and entirely sclerotised. Discoidal (discal) cell 1.6–1.7× longer than wide. Nervulus (cu-a) very short, interstitial. Mediocubital vein (M+CU1) apically almost straight. Parallel vein (CU1a) basally weakly curved. Brachial (subdiscal) cell widely open distally. Hind wing 5.6–6.0× longer than wide. First abscissa of costal vein (C+SC+R) 0.8–1.0× as long as second abscissa (1-SC+R); second abscissa (1-SC+R) sclerotised. Medial (basal) cell narrow, weakly narrowed towards apex, its length 8.0–10.0× maximum width, 0.25× length of wing. First abscissa of mediocubital vein (M+CU) 0.9–1.0× as long as second abscissa (1-M). Recurrent vein (m-cu) unsclerotised, straight, oblique, weakly antefurcal.

***Legs***. Fore tibia with several slender spines arranged in single line. Hind coxa with baso-ventral tubercle, 1.5× longer than maximum width. Hind femur rather narrow, with very low dorsal protuberance, slightly curved below (lateral view), 3.6–3.8× longer than wide. Hind tarsus 0.9× as long as hind tibia. Hind basitarsus weakly thickened, 0.40–0.45× as long as second–fifth segments combined. Second segment of hind tarsus 0.75–0.80× as long as basitarsus, 1.5× longer than fifth segment (without pretarsus).

***Metasoma***. Metasoma 2.5–2.7× longer than its maximum width, almost as long as head and mesosoma combined. First tergite with high convex median area, without visible spiracular tubercles in basal 0.3; tergite distinctly and linearly widened from base to apex. Maximum width of first tergite 2.3× its minimum basal width; its length 1.15–1.20× apical width, 1.2× length of propodeum. Suture between second and third tergites fine and smooth, weakly sinuate. Second tergite 0.6–0.7× as long as its basal width, almost as long as third tergite. Combined length of second and third tergites 1.3× basal width of second tergite, 0.8× their maximum width. Third tergite with very fine and smooth additional subbasal transverse furrow in basal third. Ovipositor sheath (measured entire length in ventrolateral view) slender, 0.5–0.6× as long as metasoma, 0.7–0.8× as long as mesosoma, 0.3× as long as fore wing.

***Sculpture and pubescence***. Vertex, frons, temple and face almost completely smooth. Mesoscutum mainly smooth, only partly very finely coriaceous, with two straight and convergent posteriorly distinct carinae along notauli, finely rugulose between them. Scutellum smooth. Mesopleuron almost entirely smooth. Propodeum with distinctly delineated, relatively long and mainly smooth baso-lateral areas, basal carina of medium length, 0.4–0.6× as long as anterior fork of areola, areola distinctly delineated, wide submedially and narrow posteriorly, pentagonal, entirely distinctly and sparsely rugose, 1.4–1.5× longer than maximum width. Hind coxa and femur mainly smooth, but coxa transverse striate dorsally. First tergite entirely distinctly and almost linearly striate and without reticulation between striae, transverse striate in baso-medial third. Second tergite entirely distinctly striate. Third and remaining tergites entirely smooth. Vertex mostly with sparse, relatively short and semi-erect pale setae, glabrous in anterior 0.3. Mesoscutum with rather sparse, medium length and almost erect pale setae arranged narrowly along notauli and in single line laterally, all lobes widely glabrous. Mesopleuron mainly glabrous. Hind tibia dorsally with medium length, rather dense and semi-erect setae; length of these setae 1.0–1.2× maximum width of hind tibia.

***Colour***. Head almost entirely black or dark brown. Mesosoma mainly dark reddish brown or reddish brown, prothorax mainly yellowish brown, mesopleuron in lower third light reddish brown. Metasoma reddish brown in basal half, light reddish brown to yellow in posterior half and below, or mainly brownish yellow. Antenna mainly brown, its two basal segments brownish yellow. Palpi pale yellow. Legs yellow, faintly infuscate basally. Ovipositor sheath dark brown. Fore wing hyaline. Pterostigma brown, yellow apically.

**Male**. Unknown.

##### Etymology.

Named after the type locality of the new species in South Korea, Chinju.

##### Distribution.

Korean Peninsula.

#### ﻿ Heterospilus (Heterospilus) extasus

Taxon classificationAnimaliaHymenopteraBraconidae

Papp, 1987

1EF31FA7-ED54-5A3E-BB02-26256203CEF0

Heterospilus extasus Papp, 1987: 163; Yu et al. 2016.

##### Additional material examined.

South Korea: 1 female, Gyeongnam-do, Sancheong-gun, Sancheong-eup, Yulhyeon-ri (Mt. Jeongsusan, Temple Yulgoksa), sweeping, 22.VIII.1998, H.-G. Ju leg.; 1 female, Gyeongbuk-do, Pohang-si, Heunhae-eup, Hakjeon-ri, (Mt. Doumsan, Temple Jeongoksa), sweeping, 17.VIII.1997, J.-S. Jeon leg.

##### Distribution.

Korean Peninsula (Papp 1987); Russia (south of Far East), China.

##### Remarks.

One female of this species (collected 17.VIII.1997) have fine to very fine and interrupted aciculation on the vertex and frons, showing thereby the intermediate state of the character between this species and *H.tirnax* Papp.

#### ﻿* Heterospilus (Heterospilus) fujianensis

Taxon classificationAnimaliaHymenopteraBraconidae

Tang, Belokobylskij, He & Chen, 2013

39E3197F-BC26-5680-87C0-8C11E72E29A2

Heterospilus fujianensis Tang, Belokobylskij, He & Chen, 2013: 218; Yu et al. 2016.

##### Material examined.

South Korea. 1 female, Gyeonggi-do, Suwon-si, Seodun-dong, Seoul National University, Agricultural College Arboretum, light trap, 18.VIII.1998, D.-S. Ku leg. (NIBR).

##### Distribution.

* Korean Peninsula; China (Jilin and Fujian Provinces).

##### Remarks.

This species is similar to *Heterospilusxanthopterus* Belokobylskij & Maetô, 2009 from Japan (Ryukyus), but differs from the latter in having the occipital carina joined ventrally with the hypostomal carina (not joined and widely separated in *H.xanthopterus*), pronotum without pronotal carina (with distinct carina in *H.xanthopterus*), notauli not wide (wide in *H.xanthopterus*), second tergite long, its median length 0.6× basal width (short, its median length 0.40–0.45× the basal width in *H.xanthopterus*), third tergite entirely smooth and without transverse subbasal furrow (with rather deep and crenulate transverse furrow in *H.xanthopterus*), vertex entirely smooth (medially striate in *H.xanthopterus*), and metasoma light reddish brown (dark reddish brown in *H.xanthopterus*).

#### ﻿ Heterospilus (Heterospilus) gajwaensis sp. nov.

Taxon classificationAnimaliaHymenopteraBraconidae

7EF315C9-E75C-5188-ADDE-60B78F3949CE

http://zoobank.org/53B5375F-15FF-4AAB-8EC9-BD73389F1C45

Figs 5 , 6

##### Type material.

***Holotype***: female, “Korea: Gyeongnam-do, Jinju-si, Gajwa-dong, 24–30. VI. 1989. Malaise trap. D.-S. Ku” (NIBR).

##### Comparative diagnosis.

This species is similar to *H.ishigakus* Belokobylskij & Maetô, 2009 from Japan (Ryukyus), but differs from the later by having the ocelli large, POL 1.3× Od and 0.7× OOL (small, POL 0.8–1.0× Od and 0.35–0.40× OOL in *H.ishigakus*), occipital carina not joined ventrally with hypostomal carina (joined at least by additional carina in *H.ishigakus*), metacarp 1.25× longer than pterostigma (1.1× in *H.ishigakus*), suture between second and third metasomal tergites weakly sinuate (distinctly sinuate in *H.ishigakus*), median length of second tergite 0.5× its basal width (0.35–0.40× in *H.ishigakus*), and ovipositor sheath shorter, 0.5× as long as metasoma and 0.3× as long as fore wing (longer, 0.8–1.0× as long as metasoma and 0.45–0.55× as long as fore wing in *H.ishigakus*).

##### Description.

**Female**. Body length 3.2 mm; fore wing length 2.5 mm.

***Head***. Head not depressed, its width (dorsal view) 1.7× median length, 1.1× width of mesoscutum. Head behind eyes (dorsal view) distinctly, regularly and roundly narrowed; transverse diameter of eye 2.6× longer than temple. Ocelli medium-sized, arranged in triangle with base 1.2× its sides. POL 1.3× Od, 0.7× OOL. Diameter of antennal socket ~ 2.0× distance between sockets, 4.0× distance between socket and eye. Eye glabrous, with shallow emargination opposite antennal sockets, 1.2× as high as broad. Malar space 0.3× height of eye, 0.8× basal width of mandible. Face weakly convex, its width 0.8× height of eye and almost equal to height of face and clypeus combined. Hypoclypeal depression rather small and round, its width almost equal to distance from edge of depression to eye, 0.5× width of face. Occipital carina complete dorsally, ventrally not reaching hypostomal carina and obliterated far before mandible base. Head below eyes (front view) rather distinctly and weakly roundly narrowed.

**Figure 5.**
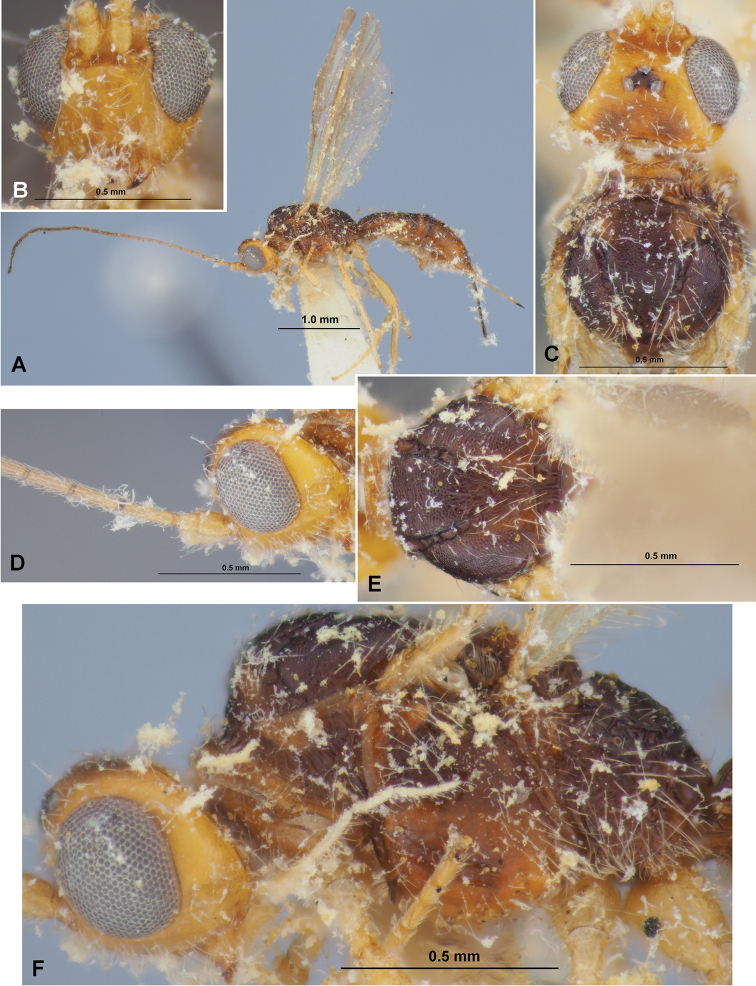
Heterospilus (Heterospilus) gajwaensis sp. nov., female, holotype **A** habitus, lateral view **B** head, front view **C** head and mesoscutum, dorsal view **D** head and basal segments of antenna, lateral view **E** mesosoma, dorsal view **F** head and mesosoma, lateral view

**Figure 6.**
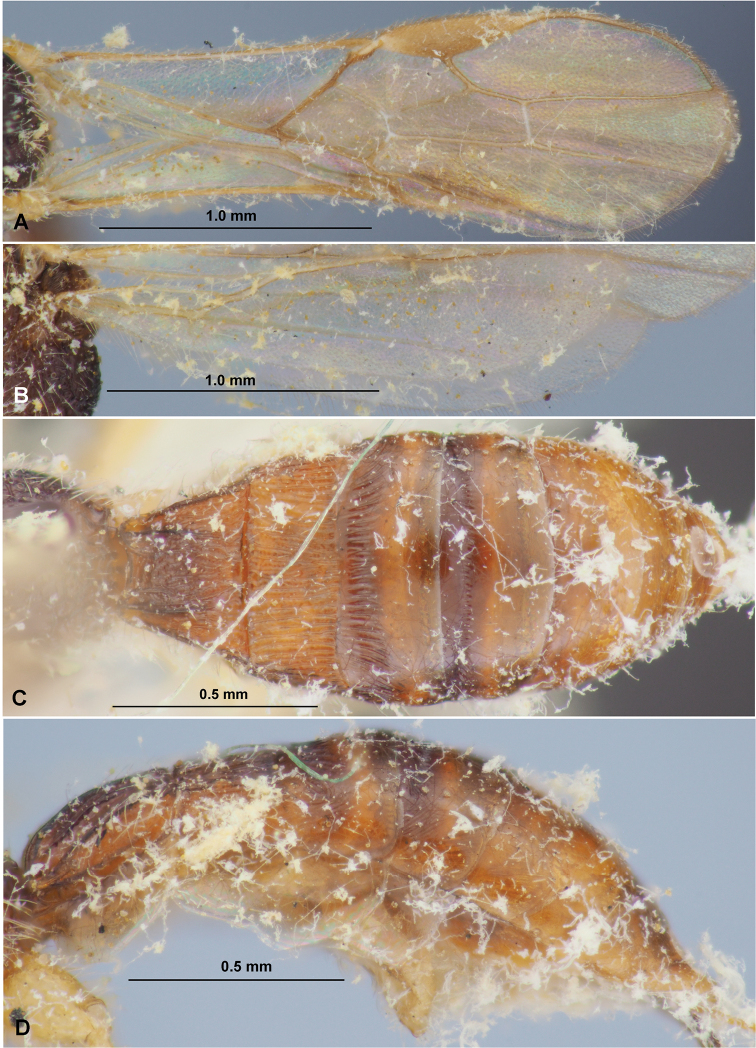
Heterospilus (Heterospilus) gajwaensis sp. nov., female, holotype **A** fore wing **B** hind wing **C** metasoma, dorsal view **D** metasoma, lateral view

***Antenna***. Antenna weakly thickened, almost filiform, 26-segmented, 1.1× longer than body. Scape rather short and thick, 1.5× longer than its maximum width. First flagellar segment weakly curved, subcylindrical, ~ 5.0× longer than its apical width, 1.05× longer than second segment. Penultimate segment ~ 3.5× longer than wide, 0.6× as long as first flagellar segment, 0.9× as long as apical segment; the latter acuminate apically and with very short spine.

***Mesosoma***. Mesosoma not depressed, its length 1.8× maximum height. Pronotum short, dorsally distinctly convex, with distinct pronotal carina submedially; side of pronotum with deep, relatively wide and distinctly curved furrow with dense crenulation. Mesoscutum highly and almost perpendicularly elevated above pronotum (lateral view), maximum width of mesoscutum (dorsal view) 1.15× its length. Median lobe of mesoscutum weakly protruding forwards, with short but distinct anterolateral corners, weakly convex anteriorly (dorsal view). Notauli relatively narrow, entirely rather deep, densely and distinctly crenulate. Prescutellar depression rather deep, wide, with three distinct carinae, entirely smooth, ~ 0.5× as long as scutellum. Scutellum weakly convex. Subalar depression rather shallow, wide, distinctly rugose-striate. Precoxal sulcus deep, straight, entirely smooth, running along anterior 0.6 of lower part of mesopleuron. Metanotal tooth very short and thick. Metapleural lobe distinct, rather narrow, rounded apically. Propodeum without lateral tubercles.

***Wings***. Fore wing 3.1× longer than its maximum width, 0.8× as long as body. Pterostigma 3.5× longer than wide. Metacarp (1-R1) 1.25× longer than pterostigma. Radial vein (r) arising weakly before middle of pterostigma, its inner distance from base of pterostigma to radial vein 0.8× distance from radial vein (r) to apex of pterostigma. First radial abscissa (r) ~ 0.9× as long as maximum width of pterostigma. Second radial abscissa (3-SR) 1.3× longer than first abscissa (r) and forming very obtuse angle with it, 0.25× as long as straight third abscissa (SR1), 0.6× as long as trace of first radiomedial vein (2-SR). Trace of first radiomedial vein (2-SR) 2.1× longer than second radiomedial vein (r-m) and 2.6× longer than recurrent vein (m-cu). Recurrent vein (m-cu) distinctly postfurcal. First medial abscissa (1-SR+M) weakly curved. Discoidal (discal) cell 1.8× longer than wide. Distance from nervulus (cu-a) to basal vein (1-M) almost equal to nervulus (cu-a) length. Mediocubital vein (M+CU1) very weakly sinuate. Parallel vein (CU1a) basally weakly curved. Brachial (subdiscal) cell widely open distally. Hind wing 4.5× longer than wide. First abscissa of costal vein (C+SC+R) 1.2× longer than second abscissa (1-SC+R); second abscissa (1-SC+R) strongly sclerotised. Medial (basal) cell narrow, almost parallel-sided in apical half, its length 7.5× maximum width, 0.25× length of wing. First abscissa of mediocubital vein (M+CU) almost as long as second abscissa (1-M). Recurrent vein (m-cu) unsclerotised, weakly curved towards apex, antefurcal.

***Legs***. Fore tibia with several slender spines arranged in almost single line. Hind coxa with distinct baso-ventral tubercle, 1.5× longer than maximum width. Hind femur rather wide, with very low dorsal protuberance, 3.6× longer than wide. Hind tarsus 0.9× as long as hind tibia. Hind basitarsus weakly thickened, 0.5× as long as second–fifth segments combined. Second segment of hind tarsus 0.75× as long as basitarsus, 1.4× longer than fifth segment (without pretarsus).

***Metasoma***. Metasoma 2.6× longer than its maximum width, 1.2× longer than head and mesosoma combined. First tergite with rather high and wide median area, without spiracular tubercles; tergite distinctly and almost linearly widened from base to apex. Maximum width of first tergite 2.2× its minimum width; its length 0.9× apical width, 1.2× length of propodeum. Second suture distinct and weakly sinuate. Median length of second tergite 0.5× its basal width, equal to length of third tergite. Combined length of second and third tergites equal to basal width of second tergite, 0.75× their maximum width. Third tergite in basal 0.3 with shallow, wide, distinctly and widely crenulate transverse furrow. Ovipositor sheath (measured entire length in ventrolateral view) relatively slender, 0.5× as long as metasoma, 0.7× as long as mesosoma, 0.3× as long as fore wing.

***Sculpture and pubescence***. Vertex entirely, distinctly and densely transversely striate, without microsculpture, without smooth spots; frons entirely densely and distinctly transversely striate. Face mainly smooth, medially and laterally in low part shortly aciculate; temple mostly smooth. Mesoscutum entirely densely and distinctly granulate, its median lobe anteriorly with granulae arranged in curved lines, with several undulate and weakly convergent posteriorly carinae and rather fine rugosity between them in its medioposterior half. Scutellum almost entirely smooth. Mesopleuron mostly smooth. Propodeum with baso-lateral areas distinctly delineated and mainly smooth with rugosity along carinae, with distinctly delineated wide pentagonal areola, basal carina short, 0.15× as long as propodeum; most part of propodeum (including areola) densely and coarsely rugose-reticulate to areolate. Hind coxae dorsally finely and only anteriorly rugulose, laterally and ventrally mainly smooth. Hind femur densely and finely striate to coriaceous in upper half, smooth on remaining part. First tergite with distinct and convergent dorsal carinae, densely, coarsely and undulately striate, with rugulosity between striae. Second tergite entirely distinctly and densely longitudinally and weakly curvedly striate, with fine micro-reticulation between striae. Third tergite densely and distinctly longitudinally striate in basal 0.3. Fourth tergite very shortly and distinctly striate in subbasal furrow. Remaining parts of tergites smooth. Vertex glabrous widely medially, with sparse, short and semi-erect setae directed forwards. Mesoscutum with rather dense, long and semi-erect white setae arranged rather widely along notauli and in single line laterally, all lobes medially widely glabrous. Mesopleuron widely glabrous medially. Hind tibia dorsally with short, rather dense and semi-erect setae; length of these setae ~ 0.5× maximum width of hind tibia.

***Colour***. Head mainly brownish yellow, faintly infuscate dorsally. Mesosoma and metasoma dark reddish brown, reddish brown to light reddish brown laterally, prothorax partly yellowish brown. Antenna dark reddish brown to black, four basal segments yellowish brown. Palpi pale yellow. Legs entirely yellow. Ovipositor sheath dark brown to black. Fore wing subhyaline. Pterostigma brown, pale brown basally and apically.

**Male**. Unknown.

##### Etymology.

Named after the type locality of the new species in South Korea, Gajwa-dong.

##### Distribution.

Korean Peninsula.

#### ﻿ Heterospilus (Heterospilus) heulriensis sp. nov.

Taxon classificationAnimaliaHymenopteraBraconidae

221A4450-C0B1-59B7-BE9A-D988FF63A14A

http://zoobank.org/D75FC087-59E3-412E-B943-5058F8B49996

Figs 7 , 8

##### Type material.

***Holotype***, female, “Korea, Gangwondo, Goseong, Ganseong, Heulri (Shinseonbong), 2.VIII–19.X.2002, D.-S. Ku, Malaise trap” (NIBR).

##### Comparative diagnosis.

This species is very similar to *H.divisus* (Wollaston, 1858), but differs from the later by having the temple shorter, transverse diameter of eye 1.5× longer than temple (longer, 1.2× longer than temple in *H.divisus*), malar space 0.75× height of eye (0.55× in *H.divisus*), mesosoma length 2.0× maximum height (1.7× in *H.divisus*), prescutellar depression with single high median carina (with three carinae in *H.divisus*), pterostigma narrow, 4.2× longer than wide (wide, 2.8× longer than wide in *H.divisus*), radial vein (r) of fore wing arising from middle of pterostigma (distinctly before in *H.divisus*), recurrent vein (m-cu) weakly antefurcal (distinctly postfurcal in *H.divisus*), hind femur narrow, 4.3× longer than wide (wide, 3.6× longer than wide in *H.divisus*), vertex and frons entirely smooth (finely and densely striate in *H.divisus*), mesoscutum finely to very finely coriaceous (distinctly granulate in *H.divisus*) and distribution in Korean Peninsula (in the south of the Western Palaearctic in *H.divisus*).

##### Description.

**Female**. Body length 1.8 mm; fore wing length 1.5 mm.

***Head***. Head not depressed, its width (dorsal view) 1.4× median length, 1.2× width of mesoscutum. Head behind eyes (dorsal view) distinctly, weakly curvedly and regularly narrowed; transverse diameter of eye 1.5× longer than temple. Ocelli small, arranged in equilateral triangle. POL 1.5× Od, 0.4× OOL. Diameter of antennal socket 0.8× distance between sockets, 2.5× distance between socket and eye. Eye without setae, with very shallow emargination opposite antennal sockets, 1.1× as high as broad. Malar space 0.75× height of eye, 1.3× basal width of mandible. Face weakly convex, its width 1.3× height of eye and 1.2× height of face and clypeus combined. Hypoclypeal depression rather small and oval, its width 0.9× distance from edge of depression to eye, 0.4× width of face. Occipital carina joined ventrally with hypostomal carina distinctly above base of mandible. Head below eyes (front view) distinctly and weakly-roundly narrowed.

**Figure 7.**
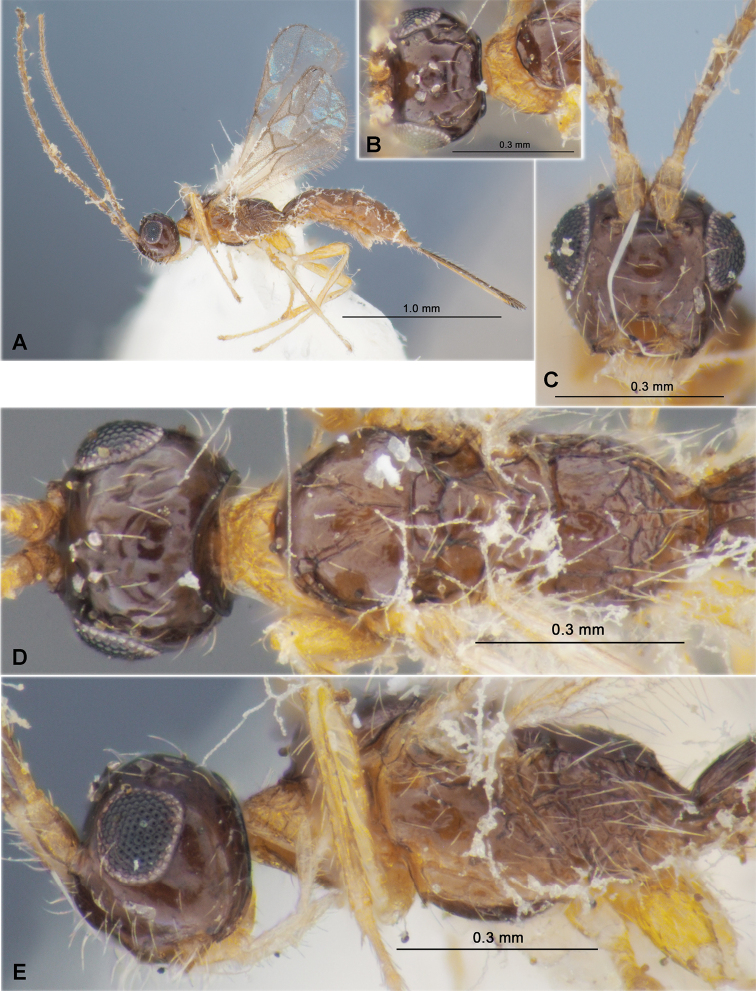
Heterospilus (Heterospilus) heulriensis sp. nov., female, holotype **A** habitus, lateral view **B** head and mesoscutum, dorsal view **C** head, front view **D** head and mesosoma, dorsal view **E** head and mesosoma, lateral view

**Figure 8.**
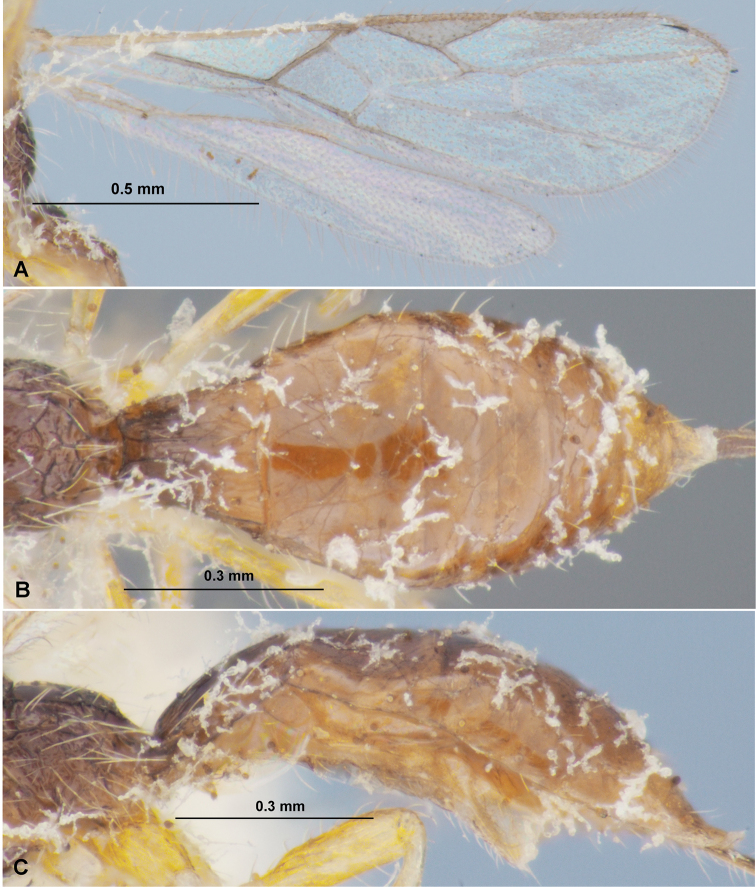
Heterospilus (Heterospilus) heulriensis sp. nov., female, holotype **A** wings **B** metasoma, dorsal view **C** metasoma, lateral view

***Antenna***. Antenna slender, filiform, 19-segmented, 1.4× longer than body. Scape short and thick, 1.3× longer than its maximum width. First flagellar segment slender, almost straight, subcylindrical, ~ 5.5× longer than its apical width, almost as long as second segment. Penultimate segment ~ 3.5× longer than wide, 0.7× as long as first flagellar segment, almost as long as apical segment; the latter obtuse apically and without spine.

***Mesosoma***. Mesosoma not depressed, its length 2.0× maximum height. Pronotum rather long, dorsally not convex (lateral view), submedially with distinct pronotal carina (dorsal view). Mesoscutum distinctly and almost perpendicularly elevated above pronotum (lateral view), maximum width of mesoscutum (dorsal view) 1.2× its length. Median lobe of mesoscutum protruding forwards, without anterolateral corners, convex anteriorly (dorsal view). Notauli narrow, deep but weakly shallow posteriorly, sparsely and finely crenulate. Prescutellar depression deep and long, with high median carina, smooth or finely rugulose, 0.4× as long as scutellum. Scutellum weakly convex, with fine lateral carinae, its basal width almost equal to median length. Subalar depression shallow, rather wide, with few coarse striae, but mainly smooth. Precoxal sulcus deep, straight, completely smooth, running along anterior 0.6 of lower part of mesopleuron. Dorsal metanotal tooth very low, wide, subpointed (lateral view). Metapleural lobe short, wide, rounded apically. Propodeum without lateral tubercles.

***Wings***. Fore wing 3.3× longer than its maximum width. Pterostigma 4.2× longer than wide. Metacarp (1-R1) 1.3× longer than pterostigma. Radial vein (r) arising from middle of pterostigma. First radial abscissa (r) almost as long as maximum width of pterostigma. Second radial abscissa (3-SR) 1.5× longer than first abscissa (r) and forming with it very obtuse angle, 0.25× as long as almost straight third abscissa (SR1), 0.65× as long as trace of first radiomedial vein (2-SR). Trace of first radiomedial vein (2-SR) ~ 2.0× longer than second radiomedial vein (r-m) and 2.5× longer than recurrent vein (m-cu). Recurrent vein (m-cu) weakly antefurcal. First medial abscissa (1-SR+M) weakly sinuate and unsclerotised. Discoidal (discal) cell elongated, almost 2.0× longer than wide. Nervulus (cu-a) short, subinterstitial. Mediocubital vein (M+CU1) apically almost straight. Parallel vein (CU1a) basally weakly curved. Brachial (subdiscal) cell widely open distally. Hind wing 6.5× longer than wide. First abscissa of costal vein (C+SC+R) almost as long as second abscissa (1-SC+R); second abscissa (1-SC+R) strongly sclerotised. Medial (basal) cell narrow, weakly narrowed towards apex, its length ~ 7.5× maximum width, 0.2× length of wing. First abscissa of mediocubital vein (M+CU) approximately as long as second abscissa (1-M). Recurrent vein (m-cu) unsclerotised, straight, subperpendicular, distinctly antefurcal.

***Legs***. Fore tibia with several distinct slender spines arranged in single line. Hind coxa with baso-ventral tubercle, 1.6× longer than maximum width. Hind femur rather narrow, with very low dorsal protuberance, slightly curved below (lateral view), 4.3× longer than wide. Hind tarsus 0.9× as long as hind tibia. Hind basitarsus weakly thickened, 0.4× as long as second–fifth segments combined. Second segment of hind tarsus 0.9× as long as basitarsus, 1.4× longer than fifth segment (without pretarsus).

***Metasoma***. Metasoma ~ 2.5× longer than its maximum width, 1.1× longer than head and mesosoma combined. First tergite with rather high but not delineated median area, with indistinct spiracular tubercles in basal 0.3; tergite distinctly and almost linearly widened from base to apex. Maximum width of first tergite 2.2× its minimum basal width; its length 1.1× apical width, 1.2× length of propodeum. Suture between second and third tergites very fine, almost indistinct. Combined length of second and third tergites 1.1× basal width of second tergite, 0.75× their maximum width. Third tergite without additional subbasal transverse furrow. Ovipositor sheath (measured entire length in ventrolateral view) slender, 0.9× as long as metasoma, 1.2× longer than mesosoma, 0.5× as long as fore wing.

***Sculpture and pubescence***. Vertex, frons, temple and face entirely smooth. Mesoscutum finely to very finely coriaceous, with two straight and convergent posteriorly distinct carinae and finely rugosity between them in narrow area in medioposterior quarter. Scutellum smooth. Mesopleuron smooth in lower 0.7. Propodeum with distinctly delineated and relatively short smooth baso-lateral areas, basal carina relatively short, 0.6× as long as anterior fork of areola, areola delineated, wide, pentagonal, entirely distinctly rugose. Hind coxa and femur smooth. First tergite entirely distinctly, strongly and almost linearly striate and without fine reticulation between striae. Second tergite almost smooth, only with very fine and short aciculation antero-laterally. Third and remainder tergites entirely smooth. Vertex with sparse, long and semi-erect pale setae. Mesoscutum with sparse, long and semi-erect pale setae arranged narrowly along notauli and in single line laterally, all lobes widely glabrous medially. Mesopleuron medially widely glabrous. Hind tibia dorsally with long, rather sparse and semi-erect setae; length of these setae almost equal to maximum width of hind tibia.

***Colour***. Head dark brown. Mesosoma dark reddish brown, prothorax mainly yellow. Metasoma dark reddish brown to reddish brown or lighter medially. Antenna mainly reddish brown, basally faintly paler. Palpi pale yellow. Legs yellow. Ovipositor sheath black. Fore wing hyaline. Pterostigma mainly yellow, partly with brownish tint.

**Male**. Unknown.

##### Etymology.

Named after the type locality of the new species in South Korea, Heulri.

##### Distribution.

Korean Peninsula.

#### ﻿ Heterospilus (Heterospilus) hyungkeunleei sp. nov.

Taxon classificationAnimaliaHymenopteraBraconidae

7FB0BA51-2A5F-5585-B0D1-BE310E18EEA4

http://zoobank.org/2C64B2A1-3B48-4D2B-8826-1ED799E93AD7

Fig. 9 , 10

##### Type material.

***Holotype***: female, “Korea: Gyeonggi-do, Osan, Sucheong-dong, Gyeonggi-do Forest Environment Research Institute, light trap, 17.IX.1999, H.-K. Lee” (NIBR).

##### Comparative diagnosis.

This species is similar to *H.nanlingensis* Tang, Belokobylskij, He & Chen, 2013, but differs from the later by having the occipital carina not joined ventrally with hypostomal carina at short distance (joined with hypostomal carina in *H.nanlingensis*), vertex smooth wide posteriorly (entirely coarsely striate in *H.nanlingensis*), mesosoma length 1.75× its maximum height (1.9× in *H.nanlingensis*), propodeum with mostly rugose-striate baso-lateral areas (mostly smooth in *H.nanlingensis*), pterostigma entirely yellow (almost entirely dark brown in *H.nanlingensis*), and suture between second and third tergites distinctly sinuate (almost straight in *H.nanlingensis*).

##### Description.

**Female**. Body length 3.2 mm; fore wing length 2.3 mm.

***Head***. Head not depressed, its width (dorsal view) 1.6× median length, 1.2× width of mesoscutum. Head behind eyes (dorsal view) distinctly convex, subparallel-sided in anterior half and distinctly roundly narrowed in posterior half; transverse diameter of eye 1.8× longer than temple. Ocelli small, arranged in almost equilateral triangle. POL almost equal to Od, 0.3× OOL. Diameter of antennal socket almost equal to distance between sockets, 2.7× distance between socket and eye. Eye glabrous, with shallow and wide emargination opposite antennal sockets, 1.2× as high as broad. Malar space 0.45× height of eye, equal to basal width of mandible. Face weakly convex, its width 1.1× height of eye and 1.1× height of face and clypeus combined. Hypoclypeal depression round, its width 0.85× distance from edge of depression to eye, 0.4× width of face. Occipital carina complete dorsally, not joined ventrally with hypostomal carina at short distance. Head below eyes (front view) distinctly and weakly curvedly narrowed.

**Figure 9.**
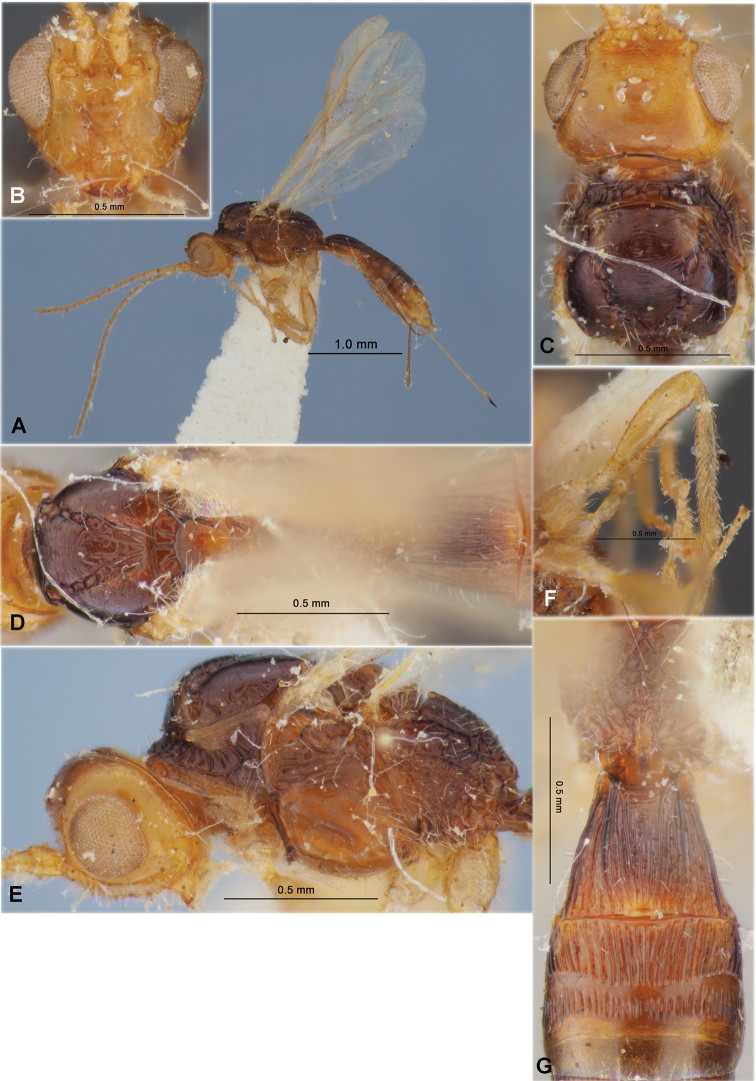
Heterospilus (Heterospilus) hyungkeunleei sp. nov., female, holotype **A** habitus, lateral view **B** head, front view **C** head and mesoscutum, dorsal view **D** mesosoma and first metasomal tergite, dorsal view **E** head and mesosoma, lateral view **F** hind leg **G** propodeum and three basal tergites of metasoma

**Figure 10.**
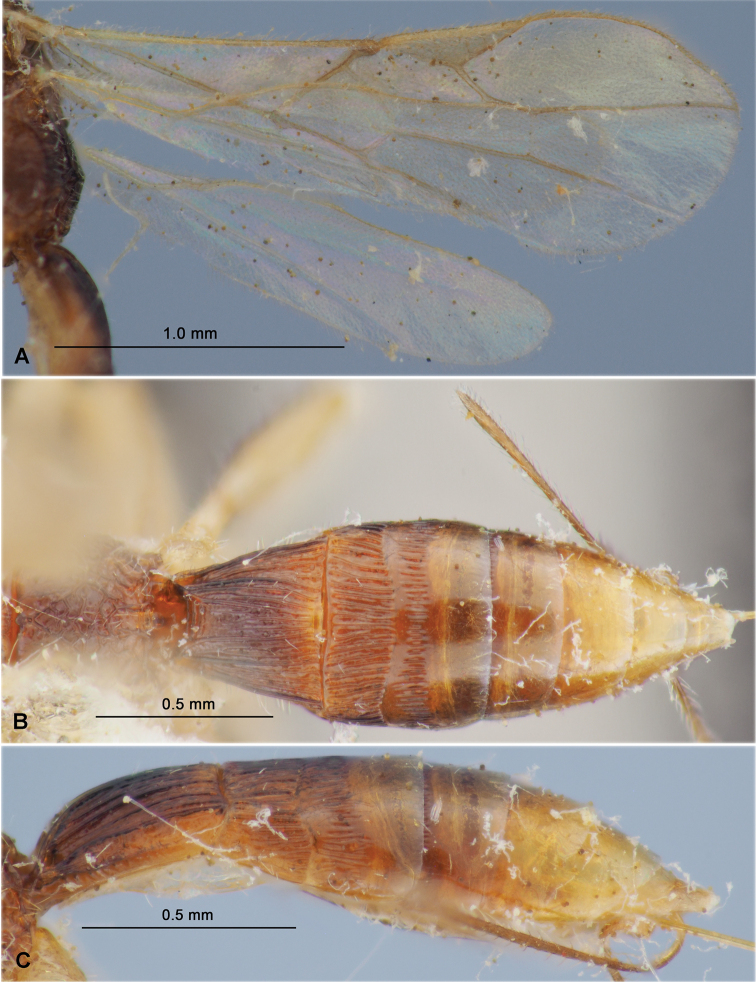
Heterospilus (Heterospilus) hyungkeunleei sp. nov., female, holotype **A** wings **B** metasoma, dorsal view **C** metasoma, lateral view

***Antenna***. Antenna slender, weakly setiform, more than 24-segmented (apical segments missing). Scape rather short and thick, 1.4× longer than its maximum width. First flagellar segment weakly thickened, almost straight, subcylindrical, 5.0× longer than its apical width, 1.2× longer than second segment. Subapical segment ~ 4.0× longer than wide.

***Mesosoma***. Mesosoma not depressed, its length 1.75× maximum height. Pronotum elongated, dorsally convex, with distinct double pronotal carina; side of pronotum with deep, rather wide, distinctly curved up and entirely coarsely crenulate furrow. Mesoscutum highly and perpendicularly elevated above pronotum (lateral view), maximum width of mesoscutum (dorsal view) 1.15× its length. Median lobe of mesoscutum (dorsal view) weakly protruding forwards, with distinct but short anterolateral corners, weakly convex anteriorly. Notauli mainly rather wide but narrowed posteriorly, coarsely and sparsely crenulate. Prescutellar depression deep, long, with three high, complete and weakly curved carinae, entirely smooth, almost 0.5× as long as scutellum. Scutellum convex, without lateral carinae, its basal width almost equal to median length. Subalar depression shallow, relatively wide, sparsely and coarsely striate. Precoxal sulcus deep, smooth anteriorly and weakly crenulate posteriorly. Propodeum without lateral tubercles.

***Wings***. Fore wing 2.9× longer than its maximum width, 0.8× as long as body. Pterostigma 3.7× longer than wide. Metacarp (1-R1) 1.5× longer than pterostigma. Radial vein (r) arising from middle of pterostigma. First radial abscissa (r) 1.1× longer than maximum width of pterostigma. Second radial abscissa (3-SR) 1.2× longer than first abscissa (r) and forming with it obtuse angle, 0.25× as long as straight third abscissa (SR1), 0.5× as long as trace of first radiomedial vein (2-SR). Trace of first radiomedial vein (2-SR) 2.1× longer than second radiomedial vein (r-m) and 2.7× longer than recurrent vein (m-cu). Recurrent vein (m-cu) postfurcal. First medial abscissa (1-SR+M) weakly sinuate. Discoidal (discal) cell 1.5× longer than wide. Distance from nervulus (cu-a) to basal vein (1-M) ~ 0.5× nervulus (cu-a) length. Mediocubital vein (M+CU1) weakly sinuate. Parallel vein (CU1a) basally weakly curved. Brachial (subdiscal) cell distally widely open. Hind wing 5.0× longer than wide. First abscissa of costal vein (C+SC+R) approximately as long as second abscissa (1-SC+R); second abscissa (1-SC+R) strongly sclerotised. Medial (basal) cell narrow, parallel-sided in apical half, its length 7.5× maximum width, 0.3× length of wing. First abscissa of mediocubital vein (M+CU) 0.9× as long as second abscissa (1-M). Recurrent vein (m-cu) unsclerotised, weakly curved towards apex of wing, weakly antefurcal.

***Legs***. Fore tibia with numerous slender spines arranged in almost straight line. Hind coxa with distinct baso-ventral tubercle, 1.3× longer than maximum width. Hind femur rather wide, with very low dorsal protuberance, 3.7× longer than wide. Hind tarsus 0.85× as long as hind tibia. Hind basitarsus weakly thickened, 0.5× as long as second–fifth segments combined. Second segment of hind tarsus 0.7× as long as basitarsus, 1.7× longer than fifth segment (without pretarsus).

***Metasoma***. Metasoma 2.8× longer than its maximum width, 1.1× longer than head and mesosoma combined. First tergite with rather high and wide median area, with almost indistinct spiracular tubercles in basal 0.3; tergite distinctly and almost linearly widened from base to apex. Maximum width of first tergite twice its minimum width; its length equal to apical width, 1.3× length of propodeum. Second suture shallow, distinct, rather distinctly sinuate. Median length of second tergite 0.3× its basal width, 0.65× length of third tergite. Combined length of second and third tergites 0.9× basal width of second tergite, 0.8× their maximum width. Third tergite with distinct and widely crenulate transverse basal furrow in anterior third. Ovipositor sheath (measured entire length in ventrolateral view) rather slender, 0.7× as long as metasoma, as long as mesosoma, 0.5× as long as fore wing.

***Sculpture and pubescence***. Vertex in anterior quarter and laterally from ocelli dense and distinctly transverse striate, smooth on remainder part. Frons entirely densely transversely striate. Temple smooth. Face mainly smooth, but finely curvedly striate medially and ventro-laterally. Mesoscutum finely transverse striate and partly with very fine granulation; scutellum entirely very finely coriaceous to smooth. Mesopleuron mostly smooth. Propodeum with mostly rugose-striate baso-lateral areas distinctly delineated by coarse carinae, with areolate-reticulate and almost completely delineated large pentagonal areola, basal carina short; most part of propodeum rather sparsely and coarsely rugose-reticulate. Hind coxae coarsely and densely transverse striate in upper half, smooth in lower half. Hind femur finely and densely reticulate-coriaceous in upper half and smooth ventrally. First tergite with distinct and convergent posteriorly dorsal carinae, rather densely and coarsely striate and with fine reticulation between striae. Second tergite entirely coarsely and sparsely striate, with very fine reticulation between striae. Third tergite mainly smooth, with widely crenulate subbasal depression in anterior third. Remainder of tergites smooth, but fourth tergite distinctly crenulate basally. Vertex mainly glabrous, with sparse and short setae marginally. Mesoscutum with relatively sparse, short and semi-erect pale setae situated narrowly only along notauli, all lobes widely glabrous. Mesopleuron widely glabrous. Hind tibia dorsally with short, relatively dense and semi-erect pale setae; length of these setae 0.4–0.5× maximum submedian width of hind tibia.

***Colour***. Head entirely brownish yellow. Mesosoma reddish brown, yellowish brown ventrally and dark reddish brown with black spots dorsally. Metasoma mainly reddish brown to dark reddish brown, distally brownish yellow. Antenna entirely brownish yellow. Palpi pale yellow. Legs entirely yellow. Ovipositor sheath dark brown. Fore wing hyaline, with faint yellowish tint. Pterostigma entirely pale yellow.

**Male**. Unknown.

##### Etymology.

Named on honour of the collector of the holotype of new species, Dr. Hyung-Keun Lee.

##### Distribution.

Korean Peninsula.

#### ﻿ Heterospilus (Heterospilus) kerzhneri

Taxon classificationAnimaliaHymenopteraBraconidae

Belokobylskij & Maetô, 2009

D149CAE6-CEE0-5128-A333-09491C25B739

Heterospilus kerzhneri Belokobylskij & Maetô, 2009: 201; Yu et al. 2016; Lee et al 2020: 19.

##### Material examined.

South Korea: 1 female, Gyeongsangnam-do, Sancheong-gun, 30 km NNW of Jinju (Chinju), forest, h = 800 m, 12.VI.2002, S. Belokobylskij leg.; 1 female, Gyeongnam, Eulryeong-Gun, Garye-nyeon, Gapeul-ri, Mt. Jengul, 12.VI.1990, D.-S. Ku leg.; 1 male, South Korea, “1987–1992”; 1 female, Kangwon, Hwachŏn, Kandong, 25.V.1993, D.-S. Ku leg.

##### Distribution.

Korean Peninsula; Russia (south of Far East), Japan.

##### Remarks.

Perhaps the specimen of *Heterospilusrubicola* Fischer, 1968 previously recorded from the Korean Peninsula as a female of *H.tobias*i Belokobylskij, 1983 (junior synonym of *H.rubicola*: Belokobylskij and Tobias, 1986) by Papp (1987) actually belongs to the morphologically similar *H.kerzhneri*. Therefore, the record of *H.rubicola* in the fauna of Korean Peninsula needs to be confirmed before it is accepted.

#### ﻿ Heterospilus (Heterospilus) maseongus sp. nov.

Taxon classificationAnimaliaHymenopteraBraconidae

F1B7856A-2515-583A-855E-FE31C32678D1

http://zoobank.org/D2A9961E-BE3D-4A32-9827-D1608C965667

Figs 11 , 12

##### Type material.

***Holotype***, female, “Korea. Kyongsangbuk-do, Chomch’on-up, Maseong Buljeong, 9.VI.1992, D.-S. Ku” (NIBR).

##### Comparative diagnosis.

This species is very similar to *H.tauricus* Telenga, 1941 and *H.indigenus* Belokobylskij, 1983 but differs from it by having the glabrous eyes (setose in both other species), recurrent vein (m-cu) interstitial (more or less postfurcal in both other species), ovipositor sheath of intermediate length, weakly longer than metasoma and 0.8× as long as fore wing (shorter, 0.5–0.6× as long as fore wing in *H.tauricus*, and longer, approximately as long as fore wing in *H.indigenus*), and mesopleuron widely smooth in lower 0.7 (densely granulate or granulate-coriaceous in both other species).

##### Description.

**Female**. Body length 4.3 mm; fore wing length 3.3 mm.

***Head***. Head not depressed, its width (dorsal view) 1.7× median length, 1.1× width of mesoscutum. Head behind eyes (dorsal view) distinctly, weakly curvedly and regularly narrowed; transverse diameter of eye 1.8× longer than temple. Ocelli medium-sized, arranged in almost equilateral triangle. POL 1.25× Od, 0.4× OOL. Diameter of antennal socket 1.1× distance between sockets, 3.0× distance between socket and eye. Eye without setae, with shallow emargination opposite antennal sockets, 1.2× as high as broad. Malar space 0.6× height of eye, almost equal to basal width of mandible. Face weakly convex, its width 1.15× height of eye and 0.9× height of face and clypeus combined. Hypoclypeal depression rather small and round, its width 0.8× distance from edge of depression to eye, 0.45× width of face. Occipital carina joined ventrally with hypostomal carina above base of mandible. Head below eyes (front view) distinctly and weakly-roundly narrowed.

**Figure 11.**
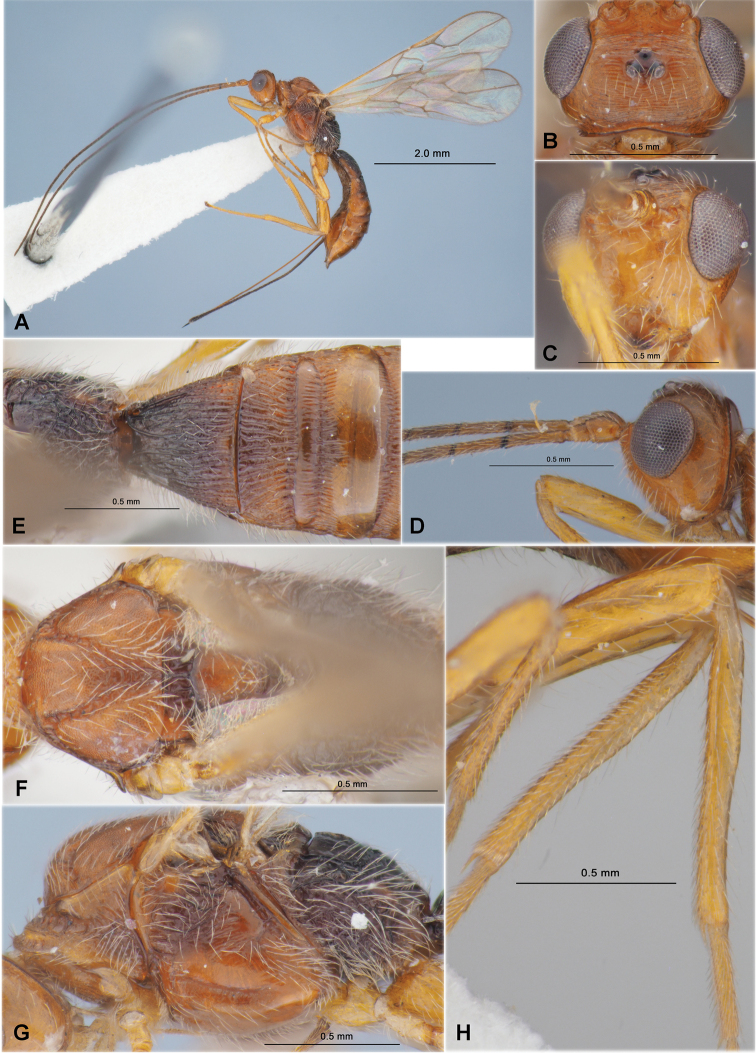
Heterospilus (Heterospilus) maseongus sp. nov., female, holotype **A** habitus, lateral view **B** head, dorsal view **C** head, front view **D** head and basal segments of antenna, lateral view **E** propodeum and three basal tergites of metasoma, dorsal view **F** mesosoma, dorsal view **G** mesosoma, lateral view **H** femur and tibia of hind leg

**Figure 12.**
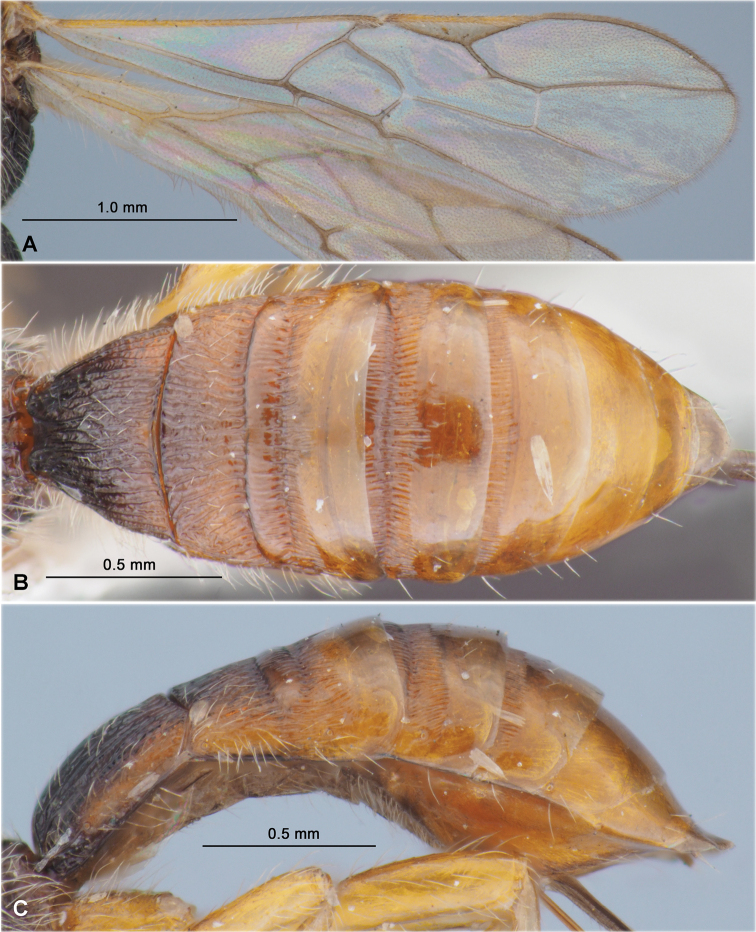
Heterospilus (Heterospilus) maseongus sp. nov., female, holotype **A** wings **B** metasoma, dorsal view **C** metasoma, lateral view

***Antenna***. Antenna slender, weakly setiform, 33-segmented, 1.3× longer than body. Scape short and thick, 1.3× longer than its maximum width. First flagellar segment rather thick, weakly curved, subcylindrical, 5.0× longer than its apical width, 1.2× longer than second segment. Penultimate segment 4.5× longer than wide, 0.55× as long as first flagellar segment, 0.9× as long as apical segment; the latter acuminate apically and without spine.

***Mesosoma***. Mesosoma not depressed, its length 1.9× maximum height. Pronotum rather long, dorsally weakly convex (lateral view), submedially with rather distinct pronotal carina (dorsal view). Mesoscutum highly and almost perpendicularly elevated above pronotum (lateral view), maximum width of mesoscutum (dorsal view) 1.1× its length. Median lobe of mesoscutum weakly protruding forwards, with small anterolateral corners, weakly convex anteriorly (dorsal view). Notauli narrow, entirely deep, sparsely and distinctly crenulate. Prescutellar depression deep, relatively long, with five distinct and weakly curved carinae, smooth between them, 0.35× as long as scutellum. Scutellum weakly convex, with fine lateral carinae, its basal width 1.1× median length. Subalar depression shallow, rather wide, coarsely striate with rugosity. Precoxal sulcus deep, almost straight, distinctly crenulate on narrow median area, running along anterior 0.6 of lower part of mesopleuron. Metanotal dorsal tooth very low, wide, subpointed (lateral view). Metapleural lobe distinct, relatively narrow, rounded apically. Propodeum without lateral tubercles.

***Wings***. Fore wing 3.5× longer than its maximum width. Pterostigma 4.8× longer than wide. Metacarp (1-R1) 1.3× longer than pterostigma. Radial vein (r) arising almost from middle of pterostigma. First radial abscissa (r) 1.2× longer than maximum width of pterostigma. Second radial abscissa (3-SR) 1.2× longer than first abscissa (r) and forming very obtuse angle with it, 0.25× as long as the straight third abscissa (SR1), 0.55× as long as trace of first radiomedial vein (2-SR). Trace of first radiomedial vein (2-SR) 2.3× longer than second radiomedial vein (r-m) and 2.5× longer than recurrent vein (m-cu). Recurrent vein (m-cu) interstitial. First medial abscissa (1-SR+M) weakly sinuate. Discoidal (discal) cell elongated, 1.8× longer than wide. Distance from nervulus (cu-a) to basal vein (1-M) 1.5× nervulus (cu-a) length. Mediocubital vein (M+CU1) apically almost straight. Parallel vein (CU1a) basally distinctly curved. Brachial (subdiscal) cell widely open distally. Hind wing 4.7× longer than wide. First abscissa of costal vein (C+SC+R) 1.6× longer than second abscissa (1-SC+R); second abscissa (1-SC+R) distinctly sclerotised. Medial (basal) cell narrow, almost parallel-sided in apical half, its length almost 9.0× maximum width, 0.25× length of wing. First abscissa of mediocubital vein (M+CU) 0.7× as long as second abscissa (1-M). Recurrent vein (m-cu) unsclerotised, straight, subperpendicular, interstitial.

***Legs***. Fore tibia with several slender spines arranged in single line. Hind coxa with distinct baso-ventral tubercle, 1.6× longer than maximum width. Hind femur rather narrow, with low dorsal protuberance, slightly curved below (lateral view), 4.4× longer than wide. Hind tarsus as long as hind tibia. Hind basitarsus weakly thickened, 0.5× as long as second–fifth segments combined. Second segment of hind tarsus 0.85× as long as basitarsus, 2.0× longer than fifth segment (without pretarsus).

***Metasoma***. Metasoma approximately 2.0× longer than its maximum width, 1.3× longer than head and mesosoma combined. First tergite with rather high but not delineated median area, with indistinct spiracular tubercles in basal 0.3; tergite distinctly and almost linearly widened from base to subapex, weakly narrowed apically. Maximum width of first tergite 2.5× its minimum basal width; its length 0.9× apical width, 1.2× length of propodeum. Suture between second and third tergites deep and finely sinuate. Median length of second tergite 0.4× its basal width, 0.7× length of third tergite. Combined length of second and third tergites 0.7× basal width of second tergite, 0.7× their maximum width. Third tergite in basal 0.25 with deep, rather wide, distinctly and widely crenulate transverse furrow. Ovipositor sheath (measured entire length in ventrolateral view) rather slender, 1.2× longer than metasoma, 1.8× longer than mesosoma, 0.8× as long as fore wing.

***Sculpture and pubescence***. Vertex and frons entirely coarsely densely and finely curvedly transversely striate, practically without additional sculpture between striae. Face finely striated laterally, medially smooth on wide area; temple smooth. Mesoscutum entirely densely and distinctly granulate, with two distinct, almost straight and convergent posteriorly carinae and with distinct rugosity between them in narrow area in its medioposterior half. Scutellum finely granulate-coriaceous. Mesopleuron widely smooth in lower 0.7, with striation in narrow transverse submedian stripe and in medioposterior area. Propodeum with distinctly delineated and short baso-lateral areas, without delineated areola, basal carina short, 0.2× as long as propodeum, 0.4× as long as anterior fork of areola; baso-lateral areas smooth in anterior half and rugulose in posterior half, remainder of propodeum densely and coarsely rugose-reticulate. Hind coxae dorsally transversely curvedly striate, laterally finely and rather densely reticulate-coriaceous. Hind femur mainly finely coriaceous. First tergite with distinct and strongly convergent subbasally dorsal carinae situated in basal half, densely, coarsely and almost linearly striate and with fine reticulation between striae. Second tergite entirely distinctly and densely longitudinally striate, and usually with reticulation between striae. Third tergite in subbasal depression widely, fourth and fifth tergites basally rather shortly and distinctly striate. Remaining parts of tergites smooth. Vertex with rather sparse, short and semi-erect setae. Mesoscutum with dense, relatively long and semi-erect pale setae arranged widely along notauli and in almost single line laterally, all lobes widely glabrous medially. Mesopleuron medially widely glabrous. Hind tibia dorsally with medium length, rather dense and semi-erect setae; length of these setae 0.5–0.7× maximum width of hind tibia.

***Colour***. Head and anterior two thirds of mesosoma reddish brown to light reddish brown. Propodeum, metapleuron and first metasomal tergite dark reddish brown to black, reminder of metasoma reddish brown to yellowish brown. Antenna dark reddish brown to black, scape, pedicel and several basal flagellar segments yellowish brown or reddish brown. Palpi yellow. Legs yellow to brownish yellow. Ovipositor sheath black. Fore wing subhyaline. Pterostigma mainly yellow with partly brownish tint.

**Male**. Unknown.

##### Etymology.

Named after the type locality of the new species in South Korea, Maseong Buljeong.

##### Distribution.

Korean Peninsula.

#### ﻿ Heterospilus (Heterospilus) separatus

Taxon classificationAnimaliaHymenopteraBraconidae

Fischer, 1960

33601B3A-A1EA-5B5F-B1DF-27A8D1A2BDF5

Heterospilus separatus Fischer, 1960: 61; Yu et al. 2016.

##### Additional material examined.

South Korea. 1 female, 3 males, Gyeongsangnam-do, Sancheong-gun, 30 km NNW of Jinju (Chinju), forest, h = 800 m, 12.VI.2002, S. Belokobylskij leg.; 2 females, Gyeonggi-do, Osan, Sucheong-dong, Gyeonggi-do Forest Environment Research Institute, light trap, 4.VI.1999, H.-G. Lee leg.; 1 female, same label, but 9.VI.1999; 1 female, Kyŏnggi, Suwon, Mt. Yŏgi, 11.V.1994, D.-S. Ku leg.

##### Distribution.

Korean Peninsula (Papp 1992); Japan, China, Russia (European part, Urals, Siberia, Far East), Mongolia, Kazakhstan, Transcaucasia, Central and Western Europe (Yu et al. 2016; Belokobylskij et al. 2019).

#### ﻿ Heterospilus (Heterospilus) suriensis sp. nov.

Taxon classificationAnimaliaHymenopteraBraconidae

50C04E15-033C-55FE-B722-1C5A49405DFE

http://zoobank.org/820120B2-3AD0-41BD-B114-4E0DE2A3A395

Figs 13 , 14

##### Type material.

***Holotype***: female, “Korea, Kyounggi, Kunpo, Sokdai, Mt. Suri, 14.VII.1998, D.-S. Ku, LT” (NIBR).

***Paratype***. 1 female, “Korea”, “1137” (SMNE).

##### Comparative diagnosis.

This new species is similar to *H.qingliangensis* Tang, Belokobylskij, He & Chen, 2013 from China (Zhejiang Province), but differs from the later by having the first flagellar segment 1.1× longer than second segment (0.9× in *H.qingliangensis*), mesoscutum almost entirely coriaceous and with rather dense setae arranged widely along notauli (almost smooth and with very sparse setae along notauli in *H.qingliangensis*), hind femur 3.6× longer than wide (4.2–4.4× in *H.qingliangensis*), suture between second and third metasomal tergites distinctly sinuate (almost straight in *H.qingliangensis*), median length of second metasomal tergite 0.45× its basal width (0.6× in *H.qingliangensis*), ovipositor sheath 0.6–0.8× as long as metasoma and 0.50–0.55 as long as fore wing (1.4 and 0.85× respectively in *H.qingliangensis*).

Also the new species is similar to *H.weolchulsanus* sp. nov., but differs from it in having the head behind eyes (dorsal view) distinctly roundly narrowed (weakly narrowed in *H.weolchulsanus*), length of first tergite equal to its apical width. (0.85× in *H.weolchulsanus*), suture between second and third metasomal tergites distinctly sinuate (very weakly sinuate in *H.weolchulsanus*), ovipositor sheath longer, 0.6–0.8× as long as metasoma and 0.50–0.55 as long as fore wing (shorter, 0.3× as long as metasoma and 0.2× as long as fore wing in *H.weolchulsanus*) and pterostigma entirely brown (light brown in *H.weolchulsanus*).

**Figure 13.**
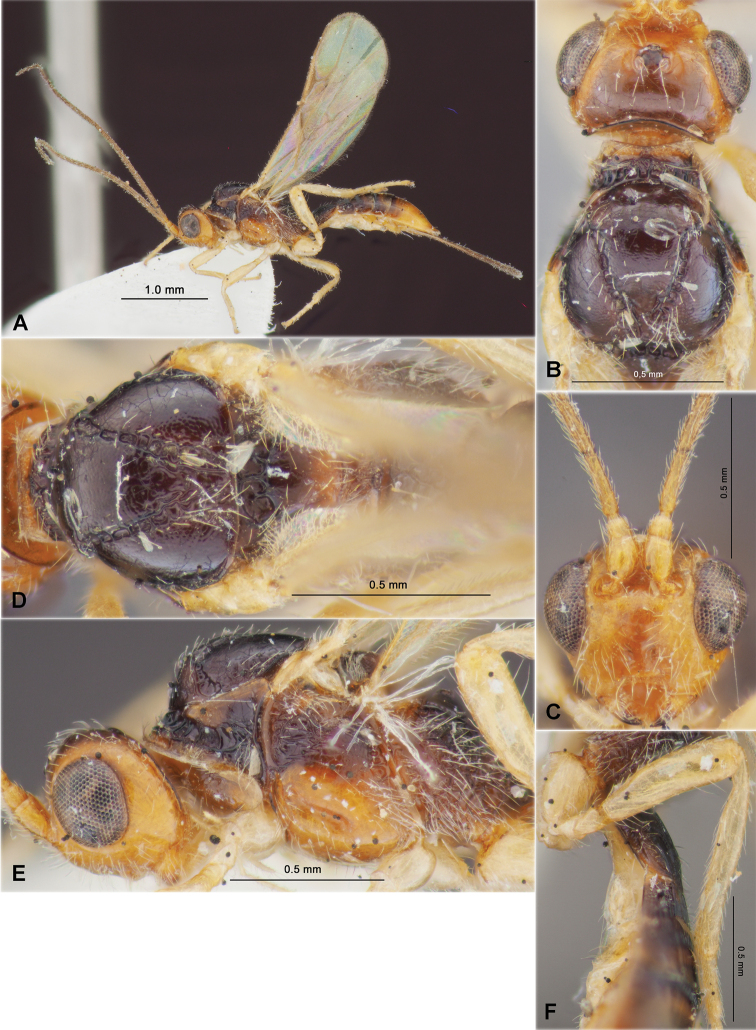
Heterospilus (Heterospilus) suriensis sp. nov., female, holotype **A** habitus, lateral view **B** head and mesoscutum, dorsal view **C** head, front view **D** mesosoma, dorsal view **E** head and mesosoma, lateral view **F** hind leg

**Figure 14.**
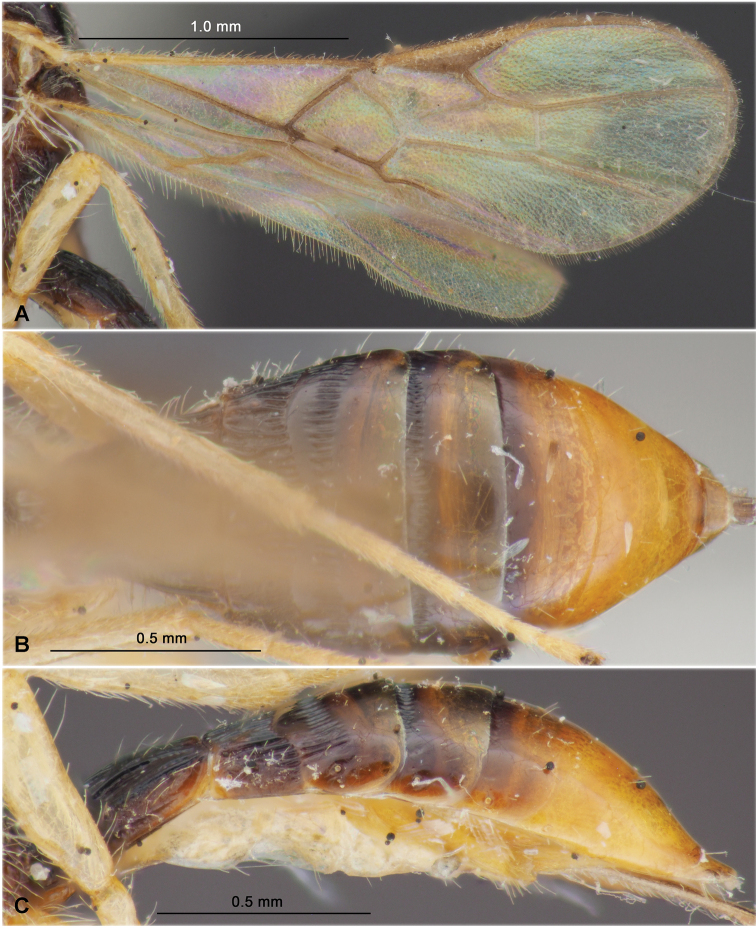
Heterospilus (Heterospilus) suriensis sp. nov., female, holotype **A** wings **B** metasoma, dorsal view **C** metasoma, lateral view

##### Description.

**Female**. Body length 2.9–3.2 mm; fore wing length 2.2–2.4 mm.

***Head***. Head not depressed, its width (dorsal view) 1.7–1.8× median length, 1.1–1.2× width of mesoscutum. Head behind eyes (dorsal view) distinctly roundly narrowed; transverse diameter of eye almost twice longer than temple. Ocelli medium-sized, arranged in triangle with base 1.1–1.2× its sides. POL 0.9–1.0× Od, 0.35–0.40× OOL. Diameter of antennal socket almost equal to distance between sockets, 2.3–2.5× distance between socket and eye. Eye glabrous, with very weak emargination opposite antennal sockets, 1.1–1.2× as high as broad. Malar space 0.45–0.50× height of eye, 1.1–1.2× basal width of mandible. Face convex, its width almost equal to height of eye and 1.1–1.2× height of face and clypeus combined. Hypoclypeal depression rather large and circular, its width almost equal to distance from edge of depression to eye, 0.45–0.50× width of face. Occipital carina complete dorsally, medially not angulate, ventrally not reaching hypostomal carina and obliterated at long distance before mandible base. Head below eyes (front view) almost linearly narrowed.

***Antenna***. Antenna rather slender, weakly setiform, 27–28-segmented, 1.2× longer than body. Scape rather long and thick, 1.4–1.5× longer than its maximum width. First flagellar segment weakly thickened, almost straight, subcylindrical, 4.8–5.0× longer than its apical width, 1.10–1.15× longer than second segment. Penultimate segment 3.0–3.5× longer than wide, 0.6–0.7× as long as first flagellar segment, 0.9–1.0× as long as apical segment, latter acuminated apically but without spine.

***Mesosoma***. Mesosoma not depressed, its length 1.8× maximum height. Pronotum short, dorsally almost flat and with distinct pronotal carina in basal 0.6, its anterior margin rather distinctly concave; side of pronotum with rather deep, relatively wide, weakly curved, coarsely and sparsely crenulate submedian oblique furrow. Mesoscutum highly and almost perpendicularly elevated above pronotum (lateral view); maximum width of mesoscutum 1.2× its length. Median lobe of mesoscutum weakly protruding forwards, with distinct obtuse anterolateral corners, very weakly convex anteriorly (dorsal view). Notauli complete, shallow, relatively wide, coarsely and sparsely crenulate. Prescutellar depression deep, relatively long, with high medial and two incomplete lateral carinae, almost smooth between carinae, 0.4× as long as scutellum. Scutellum convex, without lateral carinae, its basal width almost equal to median length. Subalar depression shallow, entirely distinctly rugose-striate. Precoxal sulcus deep, wide, straight, smooth, oblique, running along anterior half of lower part of mesopleuron. Metanotal tooth very short, wide and subpointed. Metapleural lobe short, rather wide, rounded apically. Propodeum without lateral tubercles.

***Wings***. Fore wing ~ 3.0× longer than its maximum width. Pterostigma 3.6–4.0× longer than wide. Metacarp (1-R1) 1.4–1.5× longer than pterostigma. Radial vein (r) arising almost from middle of pterostigma. First radial abscissa (r) 1.0–1.1× longer than maximum width. Second radial abscissa (3-SR) 1.3–1.4× longer than first abscissa (r), 0.25–0.30× as long as straight third abscissa (SR1), 0.5–0.7× as long as trace of first radiomedial vein (2-SR). Trace of first radiomedial vein (2-SR) 1.8–2.0× longer than second radiomedial vein (r-m) and 2.0–2.4× longer than recurrent vein (m-cu). Recurrent vein (m-cu) distinctly postfurcal. First medial abscissa (1-SR+M) distinctly curved. Discoidal (discal) cell 1.6–1.8× longer than wide. Distance from nervulus (cu-a) to basal vein (1-M) almost equal to nervulus (cu-a) length. Mediocubital vein (M+CU1) weakly sinuate. Parallel vein (CU1a) basally rather distinctly curved. Brachial (subdiscal) cell distally widely open. Hind wing 4.7–5.0× longer than wide. First abscissa of costal vein (C+SC+R) 1.0–1.3× as long as second abscissa (1-SC+R); second abscissa (1-SC+R) distinctly sclerotised. Medial (basal) cell narrow, weakly widened in apical half, its length 8.5–9.0× maximum width, 0.3× length of wing. First abscissa of mediocubital vein (M+CU) 0.7–0.8× as long as second abscissa (1-M). Recurrent vein (m-cu) unsclerotised, weakly curved towards apex, almost perpendicular to mediocubital vein, interstitial.

***Legs***. Fore tibia with numerous and slender spines densely arranged in almost single line. Hind coxa with baso-ventral tubercle, 1.4× longer than maximum width. Hind femur rather wide, with low dorsal protuberance, 3.3–3.6× longer than wide. Hind tarsus 0.9× as long as hind tibia. Hind basitarsus weakly thickened, 0.45× as long as second–fifth segments combined. Second segment of hind tarsus 0.7× as long as basitarsus, 1.3–1.5× longer than fifth segment (without pretarsus).

***Metasoma***. Metasoma 2.3–2.8× longer than its maximum width, 1.1× longer than head and mesosoma combined. First tergite with low and rather wide median area, without distinct spiracular tubercles; tergite strongly, regularly and almost linearly widened from base to apex. Maximum width of first tergite 2.3–2.5× its minimum width; its length as long as apical width, ~ 1.2× length of propodeum. Median length of second tergite 0.40–0.45× its basal width, 0.8–0.9× length of third tergite. Combined length of second and third tergites 0.9–1.1× basal width of second tergite, 0.6–0.7× their maximum width. Second suture distinct and distinctly sinuate. Third tergite in basal 0.3 with distinct crenulate transverse furrow. Ovipositor sheath (measured entire length in ventrolateral view) 0.6–0.8× as long as metasoma, 0.9–1.1× as long as mesosoma, 0.50–0.55 as long as fore wing.

***Sculpture and pubescence***. Vertex mainly smooth, only with fine short aciculation laterally of ocelli and some× in anterior third; frons mainly smooth. Face mainly smooth, with fine and short aciculation around clypeus; temple smooth. Mesoscutum finely coriaceous, partly almost smooth, with two distinctly and convergent posteriorly longitudinal carinae in medioposterior half and with fine rugosity between them. Scutellum mainly smooth, coriaceous posteriorly. Mesopleuron mainly smooth. Propodeum with baso-lateral areas distinctly delineated by high carinae, which are mainly or only in basal half smooth but widely rugose along carinae; areola distinct and pentagonal, basal carina short and situated in basal 0.2 of propodeum, remainder of propodeum coarsely rugose-reticulate. Hind coxae dorsally finely reticulate-coriaceous, or longitudinally striate in basal half, mostly smooth. Hind femur finely or very finely and densely aciculate dorsally, smooth on remainder part. First tergite densely and almost straightly longitudinally sparsely striate, with distinct additional rugosity between striae. Second tergite entirely distinctly longitudinally striate. Third tergite mainly smooth, crenulate only in narrow transverse subbasal furrow. Fourth and fifth tergites mainly smooth but crenulate in narrow basal transverse furrow (some× except fifth tergite). Remainder tergites smooth. Vertex almost entirely with sparse, short and semi-erect pale setae. Mesoscutum widely bare, with rather dense, short and semi-erect white setae situated relatively widely along notauli and in almost single line laterally. Mesopleuron medially widely glabrous. Hind tibia dorsally with short, rather dense and semi-erect pale setae; length of these setae 0.3–0.5× maximum width of hind tibia.

***Colour***. Head mainly brownish yellow, dorsally dark reddish brown or reddish brown. Mesosoma mainly dark brown to black, reddish brown or light reddish brown in lower third. Metasoma mainly dark reddish brown to black, its apical 0.25 and some× posterior halves of third–fifth tergites light brownish yellow. Antenna mainly dark brown, four–five basal segments yellow to yellowish brown. Palpi pale yellow. Legs entirely yellow. Ovipositor sheath black, dark brown basally. Fore wing weakly infuscate. Pterostigma entirely brown.

**Male** unknown.

##### Etymology.

Named after the type locality of the new species in South Korea, Mt. Suri.

##### Distribution.

Korean Peninsula.

#### ﻿ Heterospilus (Heterospilus) taehoani sp. nov.

Taxon classificationAnimaliaHymenopteraBraconidae

B2E2154F-533E-5B82-950E-2F0BCA929EF6

http://zoobank.org/BBB529E2-CCE4-41F1-BFDD-39C311D14DDE

Figs 15 , 16

##### Type material.

***Holotype***, female, “Korea, Gyeongnam-do, Jinju-si, Naedong-myeon, Doksan-ri, forest road, 1–10.VI.2003. T.-H. An, (Malaise trap)”, “HYM-BTA ATH 0000367” (NIBR).

***Paratypes***. 1 female, “Korea: GW: Yang-yang Gun: Seonyeotanggaegog, viii. [19]81, M.-J. Che”, “1041” (SMNE); 1 female, “Korea, Gyeongnam-do, Hadong-gun, Cheongam-myeon, Gunghang-ri, Jusan, 1.VI.2002. J.-S. Park (Sweeping)” (ZISP).

##### Comparative diagnosis.

This species is very similar to *H.wuyiensis* Chen & Shi, 2000 from Fujian Province of China, but differs from the latter by having the transverse diameter of eye 1.7× longer than temple (2.0× in *H.wuyiensis*), antenna 17-segmented (23-segmented in *H.wuyiensis*), first abscissa of mediocubital vein (M+CU) of hind wing 0.7× as long as second abscissa (1-M) (equal to it in *H.wuyiensis*), second metasomal tergite 0.75× as long as its basal width, and striate only in basal half (0.33× its basal width and entirely striate in *H.wuyiensis*), suture between second and third tergites present (completely absent in *H.wuyiensis*), body length 1.6 mm and fore wing length 1.4 mm (3.3 mm and 2.6 mm respectively in *H.wuyiensis*).

**Figure 15.**
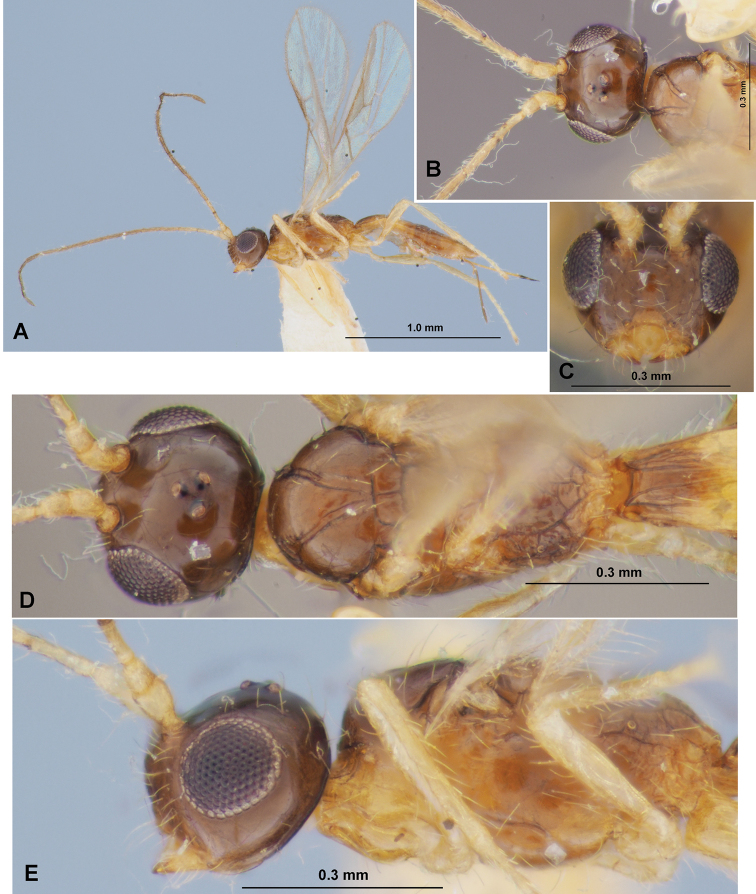
Heterospilus (Heterospilus) taehoani sp. nov., female, holotype **A** habitus, lateral view **B** basal segments of antenna, head and mesoscutum, dorsal view **C** head, front view **D** head and mesosoma, dorsal view **E** head and mesosoma, lateral view

**Figure 16.**
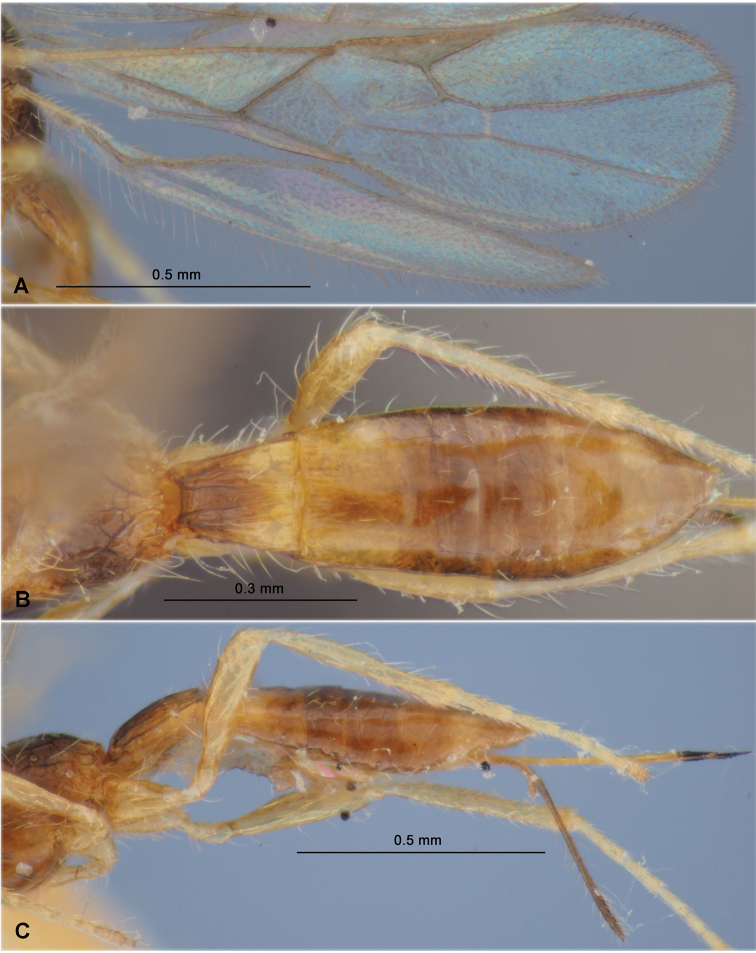
Heterospilus (Heterospilus) taehoani sp. nov., female, holotype **A** wings **B** metasoma, dorsal view **C** metasoma and ovipositor, lateral view

##### Description.

**Female**. Body length 1.6–2.1 mm; fore wing length 1.3–1.7 mm.

***Head***. Head not depressed, its width (dorsal view) 1.3–1.6× median length, 1.2–1.3× width of mesoscutum. Head behind eyes (dorsal view) regularly curvedly narrowed; transverse diameter of eye 1.4–1.5× longer than temple. Ocelli small, arranged in triangle with base 1.2× its sides. POL 1.0–1.3× Od, 0.25–0.40× OOL. Diameter of antennal socket 0.8–1.2× distance between sockets, 1.5–2.0× distance between socket and eye. Eye without setae, with very shallow emargination opposite antennal sockets, 1.2× as high as broad. Malar space 0.4–0.5× height of eye, almost equal to basal width of mandible. Face weakly convex, its width 1.0–1.2× height of eye and 1.2–1.3× height of face and clypeus combined. Hypoclypeal depression medium-sized and oval, its width 0.9–1.2× distance from edge of depression to eye, 0.4–0.5× width of face. Occipital carina joined ventrally with hypostomal carina distinctly above base of mandible. Head below eyes (front view) distinctly and roundly narrowed.

***Antenna***. Antenna slender, filiform, 16–20-segmented, 1.1–1.2× longer than body. Scape short and thick, 1.3–1.5× longer than its maximum width. First flagellar segment slender, almost straight, subcylindrical, 4.5–5.5× longer than its apical width, 0.9–1.0× as long as second segment. Penultimate segment 3.5–4.5× longer than wide, 0.8–0.9× as long as first flagellar segment and 0.9–1.0× as long as apical segment; the latter acuminate apically and without spine.

***Mesosoma***. Mesosoma not depressed, its length 1.6–1.8× maximum height. Pronotum rather long, dorsally almost not convex (lateral view), submedially with pronotal carina (dorsal view). Mesoscutum distinctly and almost perpendicularly elevated above pronotum (lateral view), maximum width of mesoscutum (dorsal view) 1.2–1.3× its length. Median lobe of mesoscutum weakly protruding forwards, without anterolateral corners, convex anteriorly (dorsal view). Notauli narrow, entirely deep, almost entirely smooth. Prescutellar depression deep and long, with high median carinae, smooth on remaining places, 0.5–0.6× as long as scutellum. Scutellum weakly convex, with fine lateral carinae, its basal width almost equal to median length. Subalar depression rather shallow, wide, with few striae medially, but smooth marginally. Precoxal sulcus deep, almost straight, entirely smooth, running along anterior 0.5 of lower part of mesopleuron. Metanotal dorsal tooth very low, wide and subpointed (lateral view). Metapleural lobe short, wide, rounded apically. Propodeum without lateral tubercles.

***Wings***. Fore wing 3.3–3.5× longer than its maximum width. Pterostigma 4.0–5.0× longer than wide. Metacarp (1-R1) 1.2–1.4× longer than pterostigma. Radial vein (r) arising from middle of pterostigma. First radial abscissa (r) 1.0–1.2× as long as maximum width of pterostigma. Second radial abscissa (3-SR) 1.7–2.2× longer than first abscissa (r) and forming with it obtuse angle, 0.2–0.4× as long as almost straight third abscissa (SR1), 0.6–0.9× as long as trace of first radiomedial vein (2-SR). Trace of first radiomedial vein (2-SR) 2.5–3.0× longer than second radiomedial vein (r-m) and 2.0–2.8× longer than recurrent vein (m-cu). Recurrent vein (m-cu) distinctly postfurcal. First medial abscissa (1-SR+M) almost straight and entirely sclerotised. Discoidal (discal) cell weakly elongated, 1.8–2.0× longer than wide. Nervulus (cu-a) very short, weakly postfurcal. Mediocubital vein (M+CU1) apically almost straight. Parallel vein (CU1a) basally very weakly curved. Brachial (subdiscal) cell widely open distally. Hind wing 6.0–6.5× longer than wide. First abscissa of costal vein (C+SC+R) ~ 0.8× as long as second abscissa (1-SC+R); second abscissa (1-SC+R) sclerotised. Medial (basal) cell narrow, weakly narrowed towards apex, its length ~ 11.0× maximum width, 0.25–0.30× length of wing. First abscissa of mediocubital vein (M+CU) 0.7–0.8× as long as second abscissa (1-M). Recurrent vein (m-cu) unsclerotised, straight, oblique, weakly antefurcal.

***Legs***. Fore tibia with several slender spines arranged in single line. Hind coxa with baso-ventral tubercle, 1.4–1.5× longer than maximum width. Hind femur rather narrow, with very low dorsal protuberance, slightly curved below (lateral view), 4.0–4.4× longer than wide. Hind tarsus 0.9–1.0× as long as hind tibia. Hind basitarsus weakly thickened, 0.4× as long as second–fifth segments combined. Second segment of hind tarsus 0.8–0.9× as long as basitarsus, 1.3× longer than fifth segment (without pretarsus).

***Metasoma***. Metasoma 3.0–3.5× longer than its maximum width, as long as head and mesosoma combined. First tergite with rather high but not delineated convex median area, without visible spiracular tubercles in basal 0.3; tergite distinctly and almost linearly widened from base to apex. Maximum width of first tergite 2.0–2.2× its minimum basal width; its length almost equal to apical width, 0.9–1.0× length of propodeum. Suture between second and third tergites fine and smooth, some× distinct only laterally or completely indistinct. Second tergite 0.5–0.7× as long as its basal width, 0.8–1.1× longer than third tergite. Combined length of second and third tergites 1.0–1.4× basal width of second tergite, 0.9–1.1× their maximum width. Third tergite with fine and smooth additional subbasal transverse furrow in basal quarter or without it. Ovipositor sheath (measured entire length in ventrolateral view) slender, 0.4–0.7× as long as metasoma, 0.7–1.0× longer than mesosoma, 0.3–0.4× as long as fore wing.

***Sculpture and pubescence***. Vertex, frons, temple and face mainly smooth, only face ventro-laterally shortly striate. Mesoscutum smooth or finely to very finely coriaceous, with two straight and convergent posteriorly distinct carinae in medioposterior half, smooth between them. Scutellum smooth. Mesopleuron almost entirely smooth in lower 0.7. Propodeum with distinctly delineated, relatively long and mainly smooth baso-lateral areas, basal carina relatively long, 0.4–0.8× as long as anterior fork of areola, areola delineated, wide anteriorly and narrow posteriorly, pentagonal, entirely distinctly rugose, 1.3–1.5× longer than maximum width. Hind coxa and femur smooth. First tergite almost entirely distinctly and almost linearly striate and without fine reticulation between striae. Second tergite distinctly striate in basal half, completely smooth in apical half. Third and remainder tergites entirely smooth. Vertex with sparse, short and semi-erect pale setae laterally and posteriorly. Mesoscutum with rather dense, long and almost erect pale setae arranged widely along notauli and in single line laterally, all lobes widely glabrous medially. Mesopleuron medially widely glabrous. Hind tibia dorsally with long, rather sparse and semi-erect setae; length of these setae 0.7–1.0× maximum width of hind tibia.

***Colour***. Head dark reddish brown. Mesosoma mainly reddish brown or light reddish brown, prothorax and some× propodeum mainly yellowish. Metasoma light reddish brown, first tergite posteriorly or rarely entirely, and some× additionally second tergite entirely and apical quarter of metasoma yellow to brownish yellow. Antenna mainly dark reddish brown, four basal segments yellow. Palpi pale. Legs yellow to pale yellow. Ovipositor sheath brown. Fore wing hyaline. Pterostigma yellow.

**Male**. Unknown.

##### Etymology.

Named on honour of the collector of the holotype of new species, Dr. Tae-Ho An.

##### Distribution.

Korean Peninsula.

#### ﻿ Heterospilus (Heterospilus) tirnax

Taxon classificationAnimaliaHymenopteraBraconidae

Papp, 1987

DC65EC33-5A69-5286-A98E-53099684ADD0

Heterospilus tirnax Papp, 1987: 163; Yu et al. 2016.

##### Additional material examined.

South Korea: 1 female, GN, Jinyang Gun: Geum Gog, 17.VII.1981, G.-J. Jeong leg.

##### Distribution.

Korean Peninsula (Papp 1987).

##### Remarks.

This specimen is characterised by the relatively long ovipositor sheath, which are 0.8× as long as metasoma and 0.6× as long as fore wing.

#### ﻿ Heterospilus (Heterospilus) weolchulsanus sp. nov.

Taxon classificationAnimaliaHymenopteraBraconidae

96314741-B58E-57DD-B96E-1DE250D21167

http://zoobank.org/E8E04589-5A7D-488F-B27D-B82508816838

Figs 17 , 18

##### Type material

. ***Holotype***: female, [South Korea] “Jeonnam-do, yeongam-gun, Gunseo-myeon, Dogap-ri, Dogapsa (Mt. Weolchulsan), sweeping, 25.VII.1999, S.-Y. Lee” (NIBR).

##### Comparative diagnosis.

This new species is similar to *H.okinawus* Belokobylskij & Maetô, 2009, but differs from the later by having the occipital carina evenly curved towards ocelli dorso-medially (angulately curved in *H.okinawus*), pronotum with distinct pronotal carina, anterior margin of pronotum distinctly concave (with fine pronotal carina, with straight anterior margin of pronotum in *H.okinawus*), maximum width of mesoscutum 1.25× its length (1.55× in *H.okinawus*), length of first metasomal tergite 0.85× its apical width (equal to its apical width in *H.okinawus*), median length of second tergite equal to length of third tergite (1.25× larger in *H.okinawus*), frons entirely smooth (finely transversely striate in *H.okinawus*), fourth tergites of metasoma smooth (basally shortly crenulate in *H.okinawus*) and pterostigma entirely light brown (brown in *H.okinawus*).

##### Description.

**Female**. Body length 3.6 mm; fore wing length 2.9 mm.

***Head***. Head not depressed, its width (dorsal view) 1.6× median length, 1.1× width of mesoscutum. Head behind eyes (dorsal view) weakly roundly narrowed; transverse diameter of eye 1.8× longer than temple. Ocelli small, arranged in almost equilateral triangle. POL almost equal to Od, 0.3× OOL. Diameter of antennal socket equal to distance between sockets, 2.3× distance between socket and eye. Eye glabrous, with very weak emargination opposite antennal sockets, 1.2× as high as broad. Malar space 0.4× height of eye, ~ 0.9× basal width of mandible. Face convex, its width 0.9× height of eye and almost equal to height of face and clypeus combined. Hypoclypeal depression rather large and subround, its width 1.1× distance from edge of depression to eye, 0.4× width of face. Occipital carina complete dorsally, medially not angulate but evenly curved towards ocelli, ventrally not reaching hypostomal carina and obliterated at long distance before mandible base. Head below eyes (front view) distinctly roundly narrowed.

**Figure 17.**
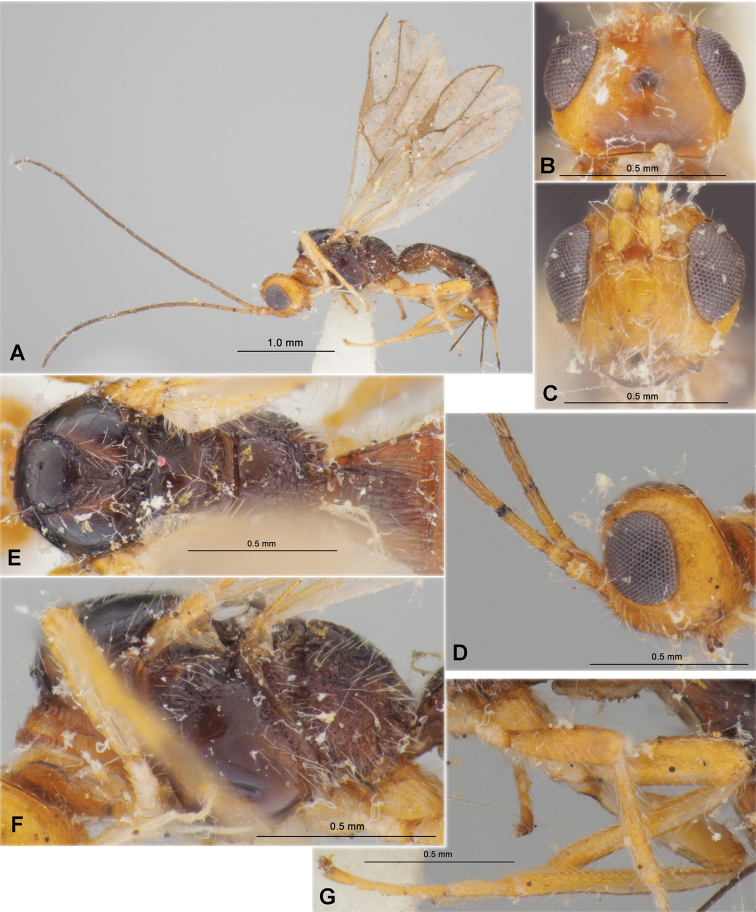
Heterospilus (Heterospilus) weolchulsanus sp. nov., female, holotype **A** habitus, lateral view **B** head, dorsal view **C** head, front view **D** head and basal segments of antenna, lateral view **E** mesosoma and first tergite of metasoma, dorsal view **F** mesosoma, lateral view **G** hind leg

**Figure 18.**
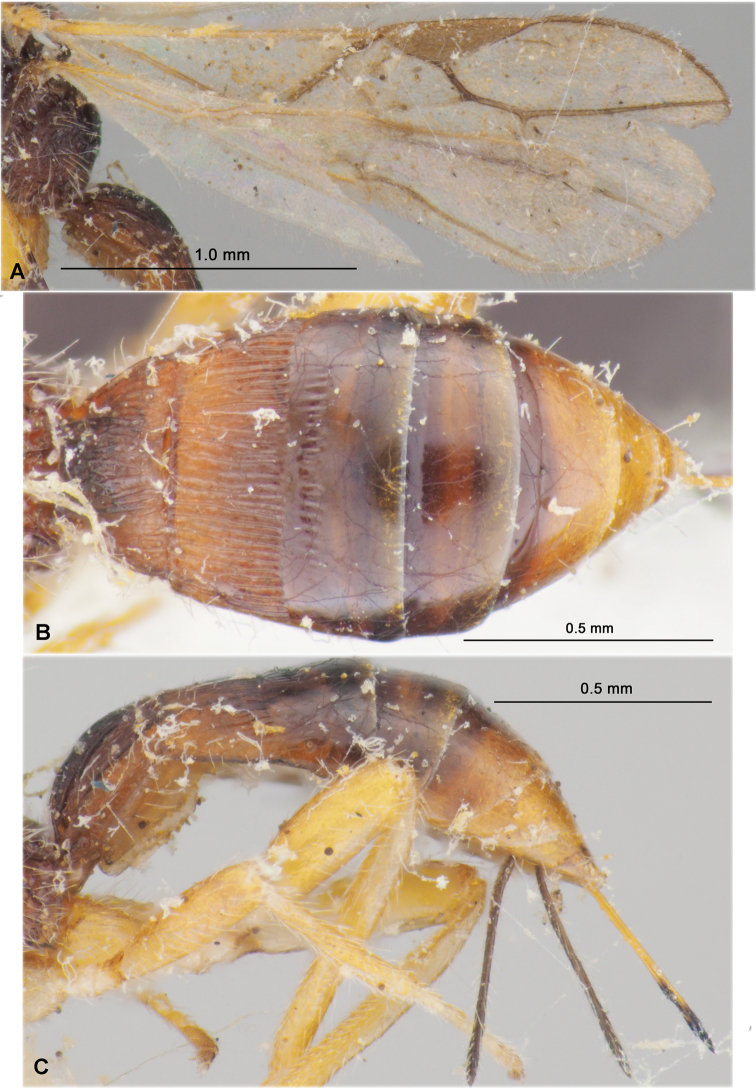
Heterospilus (Heterospilus) weolchulsanus sp. nov., female, holotype **A** wings **B** metasoma, dorsal view **C** metasoma and ovipositor, lateral view

***Antenna***. Antenna rather slender, weakly setiform, more than 23-segmented (apical segments missing). Scape short and thick, 1.6× longer than its maximum width. First flagellar segment weakly thickened, almost straight, subcylindrical, 4.3× longer than its apical width, 1.1× longer than second segment. Subapical segment ~ 5.0× longer than wide.

***Mesosoma***. Mesosoma not depressed, its length 1.7× maximum height. Pronotum short, dorsally distinctly convex and with distinct pronotal carina in basal 0.4, its anterior margin distinctly concave; side of pronotum with rather deep, narrow, almost straight and coarsely crenulate submedian oblique furrow. Mesoscutum highly and almost perpendicularly elevated above pronotum (lateral view); maximum width of mesoscutum 1.25× its length. Median lobe of mesoscutum very weakly protruding forwards, with small obtuse anterolateral corners, very weakly convex anteriorly (dorsal view). Notauli complete, rather deep, relatively wide, coarsely and sparsely crenulate. Prescutellar depression rather deep, long, with high medial and two incomplete lateral carinae, almost smooth between carinae, 0.4× as long as scutellum. Scutellum convex, without lateral carinae, its basal width almost equal to median length. Subalar depression rather deep, entirely distinctly rugose-striate. Precoxal sulcus deep, straight, smooth, oblique, running along anterior half of lower part of mesopleuron. Metanotal tooth very small, wide and subpointed. Metapleural lobe short, rather wide, rounded apically. Propodeum without lateral tubercles.

***Wings***. Fore wing 2.8× longer than its maximum width. Pterostigma 3.5× longer than wide. Metacarp (1-R1) 1.4× longer than pterostigma. Radial vein (r) arising weakly before middle of pterostigma. First radial abscissa (r) 0.9× as long as maximum width. Second radial abscissa (3-SR) 1.45× longer than first abscissa (r), 0.3× as long as straight third abscissa (SR1), 0.6× as long as trace of first radiomedial vein (2-SR). Trace of first radiomedial vein (2-SR) almost twice longer than second radiomedial vein (r-m) and 2.2× longer than recurrent vein (m-cu). Recurrent vein (m-cu) distinctly postfurcal. First medial abscissa (1-SR+M) weakly sinuate. Discoidal (discal) cell 1.5× longer than wide. Distance from nervulus (cu-a) to basal vein (1-M) 0.7× nervulus (cu-a) length. Mediocubital vein (M+CU1) almost straight. Parallel vein (CU1a) basally distinctly curved. Brachial (subdiscal) cell distally widely open. Hind wing 4.5× longer than wide. First abscissa of costal vein (C+SC+R) 1.2× longer than second abscissa (1-SC+R); second abscissa (1-SC+R) distinctly sclerotised. Medial (basal) cell narrow, weakly narrowed in apical half, its length ~ 9.0× maximum width, 0.25× length of wing. First abscissa of mediocubital vein (M+CU) as long as second abscissa (1-M). Recurrent vein (m-cu) unsclerotised, straight, almost perpendicular to mediocubital vein, interstitial.

***Legs***. Fore tibia with numerous and slender spines densely arranged in almost single line. Hind coxa with baso-ventral tubercle, 1.5× longer than maximum width. Hind femur rather wide, with low dorsal protuberance, 3.4× longer than wide. Hind tarsus 0.85× as long as hind tibia. Hind basitarsus weakly thickened, ~ 0.5× as long as second–fifth segments combined. Second segment of hind tarsus 0.6× as long as basitarsus, 1.2× longer than fifth segment (without pretarsus).

***Metasoma***. Metasoma 2.0× longer than its maximum width, almost as long as head and mesosoma combined. First tergite with high, wide and distinct median area, with very small spiracular tubercles in basal 0.3; tergite strongly, regularly and almost linearly widened from base to apex. Maximum width of first tergite 2.1× its minimum width; its length 0.85× as long as apical width, 1.2× length of propodeum. Median length of second tergite 0.5× its basal width, equal to length of third tergite. Combined length of second and third tergites almost equal to basal width of second tergite, 0.75× their maximum width. Second suture distinct and very weakly sinuate. Third tergite in basal 0.3 with shallow and distinctly crenulate transverse furrow. Ovipositor sheath (measured entire length in ventrolateral view) 0.3× as long as metasoma, 0.5× as long as mesosoma, 0.2× as long as fore wing.

***Sculpture and pubescence***. Vertex finely transverse aciculate in anterior half and smooth in posterior half; frons entirely smooth. Face almost entirely smooth; temple smooth. Mesoscutum densely and very finely coriaceous, with two distinctly convergent posteriorly longitudinal carinae in medioposterior third and distinct rugosity between them. Scutellum entirely smooth. Mesopleuron entirely smooth. Propodeum with baso-lateral areas distinctly delineated by high carinae, these areas mainly smooth but rugose along carinae; areola indistinct, basal carina short and situated in basal 0.15 of propodeum, remainder of propodeum coarsely rugose-reticulate. Hind coxae dorsally partly finely striate, mostly smooth. Hind femur finely and densely aciculate in dorsal half, almost smooth on ventral half. First tergite densely and curvedly longitudinally striate, medio-basally with additional rugosity. Second tergite entirely distinctly longitudinally striate with fine additional reticulation between striae. Third tergite crenulate only in narrow transverse subbasal furrow. Remainder tergites smooth. Vertex with sparse, short and semi-erect setae. Mesoscutum widely bare, with rather dense, long and semi-erect white setae situated widely along notauli and in single line laterally. Mesopleuron medially widely glabrous. Hind tibia dorsally with short, rather dense and semi-erect pale setae; length of these setae ~ 0.6–0.8× maximum width of hind tibia.

***Colour***. Head mainly yellow, vertex medio-posteriorly with brown spot. Mesosoma dark reddish brown, prothorax yellow anteriorly and yellowish brown posteriorly. Metasoma mainly dark reddish brown, its apical 0.25 light reddish brown. Antenna mainly black, basal segments yellow to yellowish brown. Palpi pale yellow. Legs entirely yellow. Ovipositor sheath black. Fore wing weakly infuscate. Pterostigma entirely light brown.

**Male.** Unknown.

##### Etymology.

Named after the type locality of the new species in South Korea, Mt. Weolchulsan.

##### Distribution.

Korean Peninsula.

#### ﻿ Heterospilus (Heterospilus) yeogiensis sp. nov.

Taxon classificationAnimaliaHymenopteraBraconidae

E74555B4-FAE1-5339-98E8-069AB21B05E2

http://zoobank.org/894D2108-FE6C-4EBC-B337-47A2BFAE997F

Figs 19 , 20

##### Type material.

***Holotype***: female, “Korea, Kyŏnggi, Suwon, Mt. Yeogi, 6–13.VII.1994 (M-Trap), Deok-Seo Ku” (NIBR).

##### Comparative diagnosis.

This species is very similar to *H.nishijimus* Belokobylskij & Maetô, 2008 from Ogasawara Islands of Japan, but differs from the later by having the head width (dorsal view) 1.7× its median length (1.45–1.50 in *H.nishijimus*), radial vein (r) arising before middle of pterostigma (almost from middle in *H.nishijimus*), pterostigma entirely dark brown (entirely pale brown in *H.nishijimus*), first abscissa of mediocubital vein (M+CU) of hind wing 1.3× longer than second abscissa (1-M) (0.8–1.0× in *H.nishijimus*), ovipositor sheath shorter, 0.8× as long as metasoma and 0.6× as long as fore wing (1.15–1.20× longer than metasoma and 0.75–0.85× as long as fore wing in *H.nishijimus*).

##### Description.

**Female**. Body length 3.8 mm; fore wing length 2.9 mm.

***Head***. Head not depressed, its width (dorsal view) 1.7× median length, 1.2× width of mesoscutum. Head behind eyes (dorsal view) weakly convex and roundly narrowed; transverse diameter of eye 1.8× longer than temple. Ocelli medium-sized, arranged in triangle with base 1.1× its sides. POL 1.1× Od, 0.5× OOL. Diameter of antennal socket 1.4× distance between sockets, 3.5× distance between socket and eye. Eye glabrous, with very shallow emargination opposite antennal sockets, 1.2× as high as broad. Malar space 0.35× height of eye, 0.7× basal width of mandible. Face convex, its width 0.8× height of eye and 1.1× height of face and clypeus combined. Hypoclypeal depression medium-sized and round, its width almost equal to distance from edge of depression to eye, 0.5× width of face. Occipital carina complete dorsally, ventrally not reaching hypostomal carina and obliterated at short distance before mandible base. Head below eyes (front view) distinctly and roundly narrowed.

**Figure 19.**
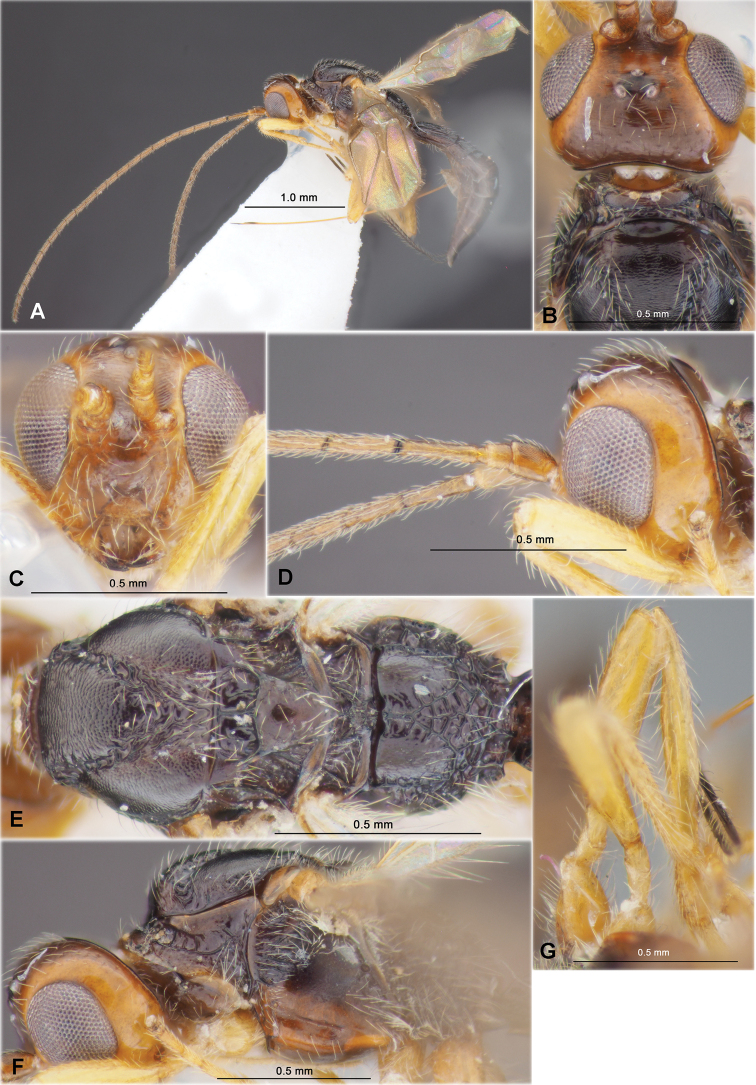
Heterospilus (Heterospilus) yeogiensis sp. nov., female, holotype **A** habitus, lateral view **B** head and mesoscutum, dorsal view **C** head, front view **D** head and basal segments of antenna, lateral view **E** mesosoma, dorsal view **F** head and mesosoma, lateral view **G** hind leg

**Figure 20.**
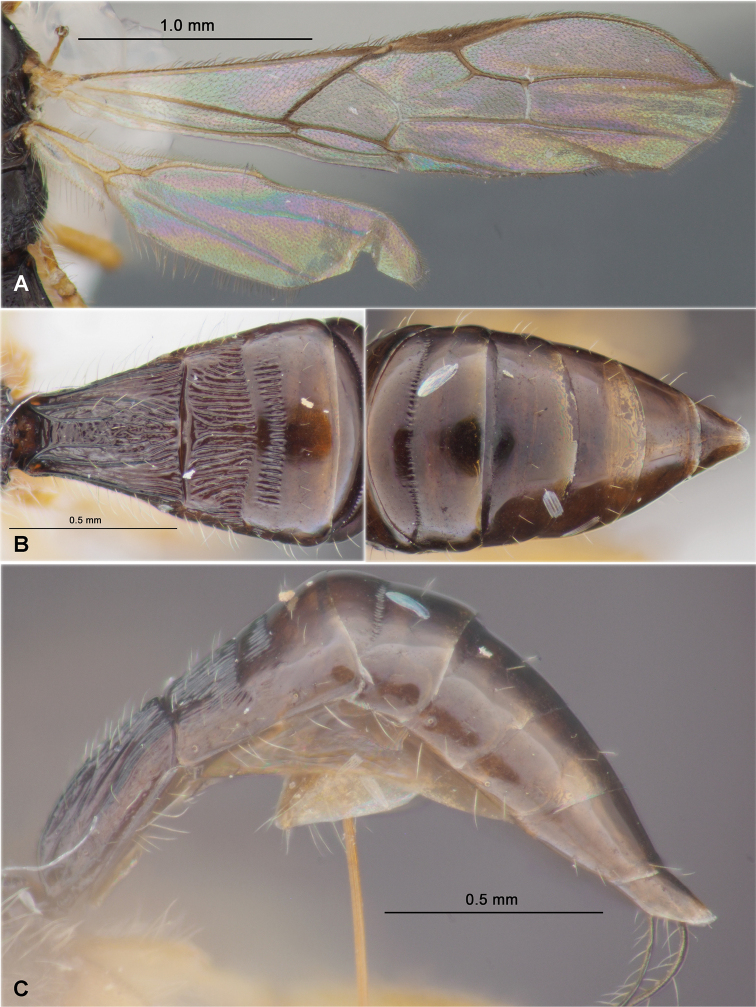
Heterospilus (Heterospilus) yeogiensis sp. nov., female, holotype **A** wings **B** metasoma, dorsal view **C** metasoma, lateral view

***Antenna***. Antenna relatively slender, setiform, 27-segmented, 1.1× longer than body. Scape rather long and thick, 1.6× longer than its maximum width. First flagellar segment slender, almost straight, subcylindrical, ~ 6.0× longer than its apical width, 1.1× longer than second segment. Penultimate segment 4.0× longer than wide, 0.6× as long as first flagellar segment, 0.9× as long as apical segment; the latter apically acuminated and without spine.

***Mesosoma***. Mesosoma not depressed, its length 1.9× maximum height. Pronotum rather long, dorsally slightly convex, with distinct submedial pronotal carina situated in posterior third; side of pronotum with rather deep, narrow, weakly curved and sparsely crenulate submedian furrow. Mesoscutum highly and almost perpendicularly elevated above pronotum (lateral view), maximum width of mesoscutum (dorsal view) equal to its length. Median lobe of mesoscutum distinctly protruding forwards, with distinct anterolateral corners, slightly convex anteriorly (dorsal view). Notauli entirely wide and deep, sparsely and distinctly crenulate. Prescutellar depression rather deep, long, with three carinae, mostly smooth, 0.4× as long as wide, 0.5× as long as scutellum. Scutellum slightly convex, without lateral carinae, its basal width 1.1× median length. Subalar depression shallow, rather wide, coarsely rugose-striate. Precoxal sulcus rather deep, straight, slightly oblique, completely smooth, running along anterior 0.6 of lower part of mesopleuron. Metanotal tooth short and angulated. Metapleural lobe long, narrow, rounded apically. Propodeum without lateral tubercles.

***Wings***. Fore wing 3.2× longer than its maximum width, 0.7× as long as body. Pterostigma 3.8× longer than wide. Metacarp (1-R1) 1.3× longer than pterostigma. Radial vein (r) arising before middle of pterostigma, its basal inner margin 0.7× as long as apical inner margin First radial abscissa (r) 0.8× as long as maximum width of pterostigma. Second radial abscissa (3-SR) 1.6× longer than first abscissa (r) and forming with it very obtuse angle, 0.25× as long as straight third abscissa (SR1), 0.7× as long as trace of first radiomedial vein (2-SR). Trace of first radiomedial vein (2-SR) 2.6× longer than second radiomedial vein (r-m) and 2.6× longer than recurrent vein (m-cu). Recurrent vein (m-cu) distinctly postfurcal. First medial abscissa (1-SR+M) straight. Discoidal (discal) cell 1.8× longer than wide. Posterior abscissa of basal vein (1-M) 2.8× longer than recurrent vein (m-cu). Distance from nervulus (cu-a) to basal vein (1-M) 1.3× nervulus (cu-a) length. Mediocubital vein (M+CU1) almost straight. Parallel vein (CU1a) basally weakly curved. Brachial (subdiscal) cell widely open distally. Hind wing 5.0× longer than wide. First abscissa of costal vein (C+SC+R) 1.5× longer than second abscissa (1-SC+R); second abscissa (1-SC+R) strongly sclerotised. Medial (basal) cell narrow, almost parallel-sided in apical half, its length 10.0× maximum width, ~ 0.3× length of wing. First abscissa of mediocubital vein (M+CU) 1.3× longer than second abscissa (1-M). Recurrent vein (m-cu) sclerotised basally, unsclerotised apically, weakly curved, oblique towards apex of wing, interstitial.

***Legs***. Fore tibia with several slender spines arranged in narrow stripe. Hind coxa with distinct baso-ventral tubercle, 1.6× longer than maximum width. Hind femur rather narrow, without distinct dorsal protuberance, almost 4.0× longer than wide.

***Metasoma***. Metasoma 3.3× longer than its maximum width, 1.3× longer than head and mesosoma combined. First tergite with distinct and rather narrow median area, without spiracular tubercles; tergite distinctly and linearly widened from base to apex. Maximum width of first tergite 2.1× its minimum width; its length 1.2× apical width, 1.3× length of propodeum. Median length of second tergite 0.4× its basal width, 0.8× length of third tergite. Combined length of second and third tergites 0.9× basal width of second tergite, 0.7× their maximum width. Second suture distinct, narrow, not curved laterally. Third tergite in basal 0.3 medially widely with shallow and distinctly crenulate transverse furrow widened medially. Ovipositor sheath (measured its entire length in ventrolateral view) slender, 0.8× as long as metasoma, 1.4× longer than mesosoma, 0.6× as long as fore wing.

***Sculpture and pubescence***. Vertex finely and densely interruptedly transversely striate and without additional microsculpture in anterior half, smooth in posterior half; frons almost entirely densely and curvedly transversely striate. Face almost entirely smooth, partly with fine punctation; temple smooth. Scape finely and densely coriaceous in upper half. Mesoscutum entirely distinctly and densely granulate-reticulate, granulae situated in transverse dense lines in anterior half of median lobe, with two curved and convergent posteriorly distinct carinae, with rugulose area in medio-posterior half. Scutellum smooth. Mesopleuron mainly smooth on wide area. Propodeum with distinctly delineated and narrowed posteriorly baso-lateral areas, areola distinctly delineated and narrow, entirely coarsely reticulate-rugose; basal carina short, 0.25× as long as propodeum; baso-lateral areas coarsely rugulose along carinae, finely coriaceous to smooth on remaining part, remainder of propodeum coarsely rugose-reticulate. Hind coxae entirely smooth. Hind femur very finely and densely aciculate dorsally, smooth on remaining part. First tergite with distinct and posteriorly convergent dorsal carinae; densely, coarsely and curvedly striate, distinctly rugose-reticulate medially, basally transversely striate. Second tergite entirely distinctly longitudinally striate, striae subparallel, medially with fine microsculpture. Third tergite distinctly crenulate in subbasal transverse furrow. Subbasal transverse furrow of fourth tergite finely striate at very short area. Remaining parts of tergites smooth. Vertex mainly with sparse, relatively long and semi-erect pale setae, glabrous anteriorly and laterally. Mesoscutum with more or less dense, rather long and almost erect pale setae arranged widely along notauli and almost in single line laterally, all lobes medially widely glabrous. Mesopleuron medially widely glabrous. Hind tibia dorsally with medium length, rather dense and semi-erect pale setae; length of these setae 0.5–0.7× maximum width of hind tibia.

***Colour***. Head mainly dark brown, around eyes with yellow stripes widened posteriorly. Mesosoma and metasoma mainly black, mesopleuron reddish brown in lower half. Antenna mainly black, dark reddish brown basally. Palpi yellow. Legs mainly yellow, hind coxa light reddish brown. Ovipositor sheath evenly black. Fore wing very faintly infuscate. Pterostigma entirely dark brown.

**Male**. Unknown.

##### Etymology.

Named after the type locality of the new species, Mt. Yŏgi.

##### Distribution.

Korean Peninsula.

### ﻿Key to *Heterospilus* species found on the Korean peninsula

**Table:** 

1	Ventral margin of scape not shorter than its dorsal margin. Second abscissa of costal vein (1-SC+R) of hind wing absent (Subgenus Eoheterospilus Belokobylskij & Maetô, 2009)	**H. (E.) rubrocinctus (Ashmead, 1905)**
–	Ventral margin of scape shorter than its dorsal margin. Second abscissa of costal vein (1-SC+R) of hind wing always present. (Subgenus Heterospilus Haliday, 1836)...................	.........**2**
2	Mesoscutum entirely smooth or some× finely to very finely coriaceous (Figs 3D, 7D, 13D, 15D, 17E)	**3**
–	Mesoscutum distinctly and densely granulate, rarely with at least fine semi-circular striation on anterior half of middle lobe (Figs 1E, 5E, 9D, 11F, 19E)	**11**
3	Second metasomal tergite almost entirely smooth or striate only basally (Figs 8B, 16B). Suture between second and third tergites very fine, almost indistinct at least medially (Figs 8B, 16B). – Pterostigma yellow (Figs 8A, 16A)	**4**
–	Second metasomal tergite entirely sculptured, striate and some× with additional reticulation (Figs 4B, 14B, 18B). Suture between second and third tergites distinct and complete (Figs 4B, 14B, 18B)	**5**
4	Second metasomal tergite entirely smooth (Fig. 8B). Combined length of second and third tergites 0.75× their maximum width (Fig. 8B). Third tergite without subbasal transverse furrow (Fig. 8B). Ovipositor sheath long, 0.9× as long as metasoma, 0.5× as long as fore wing (Fig. 7A). Body length 1.8 mm	***H.heulriensis* sp. nov.**
–	Second metasomal tergite distinctly striate in basal half, completely smooth in apical half (Fig. 16B). Combined length of second and third tergites 0.9–1.1× their maximum width (Fig. 16B). Third tergite usually with fine and smooth subbasal transverse furrow (Fig. 16B). Ovipositor sheath short, 0.4–0.7× as long as metasoma, 0.3–0.4× as long as fore wing (Fig. 15A). Body length 1.6–2.1 mm	***H.taehoani* sp. nov.**
5	Ovipositor sheath 0.8–1.2, rarely almost 2.0, × as long as metasoma, 1.2–1.8, rarely almost 3.0, × longer than mesosoma, 0.5–0.8, rarely 1.3–1.5, × as long as fore wing (Fig. 13A)	**6**
–	Ovipositor sheath 0.3–0.6× as long as metasoma, 0.5–0.9× as long as mesosoma, 0.2–0.4× as long as fore wing (Figs 3A, 17A)	**8**
6	Ovipositor sheath almost 2.0× longer than metasoma, 2.5–3.0× longer than mesosoma, 1.3–1.5× longer than fore wing. Body length 3.5–5.0 mm	***H.zaykovi* van Achterberg, 1992**
–	Ovipositor sheath 0.8–1.2× as long as metasoma, 1.2–1.8× longer than mesosoma, 0.5–0.8× as long as fore wing	**7**
7	Mesoscutum almost entirely smooth. Suture between second and third metasomal tergites almost straight. Pterostigma dark brown. Third tergite usually smooth subbasally. Body length 1.7–4.3 mm	***H.separatus* Fischer, 1960**
–	Mesoscutum almost entirely coriaceous (Fig. 13D). Suture between second and third metasomal tergites distinctly sinuate (Fig. 14B). Pterostigma yellow or pale brown (Fig. 14A). Third tergite crenulate subbasally (Fig. 13D). Body length 2.9–3.2 mm	***H.suriensis* sp. nov.**
8(5)	First metasomal tergite narrow, its length 1.15× distal width (Figs 3E, 4B). Third tergite without subbasal transverse furrow (Fig. 4B). Body length 2.0 mm	***H.chinjuensis* sp. nov.**
–	First metasomal tergite relatively wide, its length not larger than distal width (Fig. 17E). Third tergite usually (except *H.fujianensis*) with crenulate subbasal transverse furrow (Fig. 18B)	**9**
9	Mesosoma short, its length 1.7× maximum height (Fig. 17F). Maximum width of mesoscutum 1.25× its median length (Fig. 17E). Head mostly yellow, mesosoma and metasoma contrastingly dark brown (Fig. 17A). Body length 3.6 mm	***H.weolchulsanus* sp. nov.**
–	Mesosoma long, its length 1.8–2.0× maximum height. Maximum width of mesoscutum 1.10–1.15× its median length. Head dark or yellow, mesosoma and metasoma often of same colour as head or pale brown	**10**
10	Third metasomal tergite with striate transverse subbasal furrow. Body often mostly pale. Body length 1.6–3.1 mm	***H.chinensis* Chen & Shi, 2004**
–	Third metasomal tergite without striate transverse furrow. Usually head dark and remainder of body pale brown. Body length 2.5 mm	***H.fujianensis* Tang, Belokobylskij, He & Chen, 2013**
11(2)	Fourth metasomal tergite basally always more or less distinctly striate, often fifth tergite also basally striate (Figs 6C, 10B, 12B)	**12**
–	Fourth and fifth metasomal tergite basally always smooth (Figs 2C, 20B)	**19**
12	Mesosoma depressed, its length 2.2–2.7× maximum height. Malar space 0.3–0.4× height of eye, 0.7–0.9× basal width of mandible. Median length of second tergite 0.55–0.60× its basal width, 1.20–1.35× length of third tergite. Body length 2.4–3.1 mm	***H.kerzhneri* Belokobylskij & Maetô, 2009**
–	Mesosoma not depressed, its length 1.8–2.0× maximum height (Figs 5F, 9E, 11G). Malar space 0.50–0.65× height of eye, 1.1–1.3× basal width of mandible (Figs 5B, 9B, 11C) . Median length of second tergite 0.3–0.4× its basal width, 0.5–0.8× length of third tergite (Figs 6C, 10B, 12B)	**13**
13	Transverse diameter of eye 2.6× longer than temple (dorsal view) (Fig. 5C). Median length of second tergite 0.5× its basal width (Fig. 6C). Body length 3.2 mm	***H.gajwaensis* sp. nov.**
–	Transverse diameter of eye 1.5–1.7× longer than temple (dorsal view) (Figs 9C, 11B). Median length of second tergite 0.3–0.4× its basal width (Figs 10B, 12B)	**14**
14	Vertex mainly smooth (Fig. 9C). Median lobe of mesoscutum finely transverse striate anteriorly (Fig. 9D). Suture between second and third tergites distinctly sinuate (Figs 9G, 10B). Body length 3.2 mm	***H.hyungkeunleei* sp. nov.**
–	Vertex entirely strongly transverse striate (Fig. 11B). Median lobe of mesoscutum only distinctly granulate anteriorly, without striation (Fig. 11F). Suture between second and third tergites straight or some× only weakly sinuate (Fig. 12B)	**15**
15	Fifth metasomal tergite always smooth basally. Length of first metasomal tergite 1.0–1.1× its apical width. Ovipositor sheath thickened, shorter, 0.6–0.8× as long as metasoma and 0.4–0.5× as long as fore wing. – Trace of recurrent vein (m-cu) antefurcal. Body length 1.7–2.9 mm	***H.leptosoma* Fischer, 1960**
–	Fifth metasomal tergite always striate basally (Figs 12B, 12C). Length of first metasomal tergite usually 0.8–0.9 (rarely 1.0) × its apical width (Figs 11E, 12B). Ovipositor sheath not thickened, long (except *H.cephi*), as long as or longer than metasoma and 0.6–0.7× as long as fore wing (Fig. 11A)	**16**
16	Eye with short and sparse setae. Recurrent vein (m-cu) distinctly postfurcal to trace of first radiomedial vein (2-SR). Length of first metasomal tergite usually 0.8–0.9× its apical width	**17**
–	Eye glabrous (Fig. 5B). Recurrent vein (m-cu) interstitial to trace of first radiomedial vein (2-SR) (Fig. 12A). Length of first metasomal tergite usually not shorter than its apical width	**18**
17	Ovipositor sheath 0.4–0.6× as long as metasoma, shorter than mesosoma, 0.3–0.4× as long as fore wing. Body often entirely yellow or yellowish brown. Body length 2.0–4.1 mm	***H.cephi* Rohwer, 1925**
–	Ovipositor sheath 0.7–1.0× as long as metasoma, equal to or longer than mesosoma, 0.4–0.7× as long as fore wing. Body dark reddish brown to black or light reddish brown with dark propodeum and first tergite. Body length 1.9–3.9 mm	***H.tauricus* Telenga, 1941**
18	Length of first metasomal tergite 1.0-1.1× its apical width. Ovipositor sheath ~ as long as metasoma, 1.2× longer than mesosoma, 0.6× as long as fore wing. Body almost entirely brownish yellow. Body length 2.8–3.0 mm	***H.tirnax* Papp, 1987**
–	Length of first metasomal tergite 0.9× its apical width (Fig. 11E). Ovipositor sheath 1.2× longer than metasoma, 1.8× longer than mesosoma, 0.8× as long as fore wing (Fig. 11A). Body different colour; head and anterior two thirds of mesosoma reddish brown to light reddish brown; propodeum and metapleuron of mesosoma and first metasomal tergite dark reddish brown to black, remainder of metasoma reddish brown to yellowish brown (Fig. 11A). Body length 4.3 mm	***H.maseongus* sp. nov.**
19(11)	Ovipositor sheath 0.3–0.4× as long as metasoma, 0.5–0.6× as long as mesosoma, 0.2–0.3× as long as fore wing (Fig. 1A). – Subbasal transverse furrow on third metasomal tergite usually present, but rarely it fine or almost absent (Fig. 1G). Body length 1.5–3.5 mm	***H.austriacus* (Szepligeti, 1906) (= *H.ater* Fischer, 1960)**
–	Ovipositor sheath 0.8–1.0× as long as metasoma, 1.0–1.5× longer than mesosoma, 0.4–0.7× as long as fore wing (Fig. 19A)	**20**
20	Pterostigma dark brown (Fig. 20A). Length of first metasomal tergite 1.2× its apical width (Fig. 20B). Radial vein (r) arising before middle of pterostigma (Fig. 20A). Suture between second and third tergites sinuate (Fig. 20B). – Ovipositor sheath 0.8× as long as metasoma, 0.6× as long as fore wing (Fig. 19A). Body length 3.8 mm	***H.yeogiensis* sp. nov.**
–	Pterostigma pale brown or yellow. Length of first metasomal tergite not large than its apical width. Radial vein (r) arising from middle of pterostigma. Suture between second and third tergites almost straight	**21**
21	Head behind and below eyes almost linearly narrowed. Body in long and suberect setae, mainly dark. Body length 2.0–3.4 mm	***H.orientalis* Belokobylskij, 1983**
–	Head behind and below eyes roundly narrowed. Body in short and semi-erect setae, mainly pale. Body length 2.3 mm	***H.extasus* Papp, 1987**

## Supplementary Material

XML Treatment for Heterospilus (Heterospilus) austriacus

XML Treatment for Heterospilus (Heterospilus) chinensis

XML Treatment for Heterospilus (Heterospilus) chinjuensis

XML Treatment for Heterospilus (Heterospilus) extasus

XML Treatment for Heterospilus (Heterospilus) fujianensis

XML Treatment for Heterospilus (Heterospilus) gajwaensis

XML Treatment for Heterospilus (Heterospilus) heulriensis

XML Treatment for Heterospilus (Heterospilus) hyungkeunleei

XML Treatment for Heterospilus (Heterospilus) kerzhneri

XML Treatment for Heterospilus (Heterospilus) maseongus

XML Treatment for Heterospilus (Heterospilus) separatus

XML Treatment for Heterospilus (Heterospilus) suriensis

XML Treatment for Heterospilus (Heterospilus) taehoani

XML Treatment for Heterospilus (Heterospilus) tirnax

XML Treatment for Heterospilus (Heterospilus) weolchulsanus

XML Treatment for Heterospilus (Heterospilus) yeogiensis
